# Acoustic pipette and biofunctional elastomeric microparticle system for rapid picomolar-level biomolecule detection in whole blood

**DOI:** 10.1126/sciadv.ado9018

**Published:** 2024-10-16

**Authors:** Cooper P. Thome, John P. Fowle, Parker McDonnell, Johanna Zultak, Kaushik Jayaram, Aaron K. Neumann, Gabriel P. López, C. Wyatt Shields

**Affiliations:** ^1^ Department of Chemical and Biological Engineering, University of Colorado Boulder, Boulder, CO 80303, USA.; ^2^ Paul M. Rady Department of Mechanical Engineering, University of Colorado Boulder, Boulder, CO 80309, USA.; ^3^ Biomedical Engineering Program, University of Colorado Boulder, Boulder, CO 80303, USA.; ^4^ Department of Pathology, University of New Mexico School of Medicine, Albuquerque, NM 87131, USA.; ^5^ Department of Chemical and Biological Engineering, University of New Mexico, Albuquerque, NM 87131, USA.

## Abstract

Most biosensing techniques require complex processing steps that generate prolonged workflows and introduce potential points of error. Here, we report an acoustic pipette to purify and label biomarkers in 70 minutes. A key aspect of this technology is the use of functional negative acoustic contrast particles (fNACPs), which display biorecognition motifs for the specific capture of biomarkers from whole blood. Because of their large size and compressibility, the fNACPs robustly trap along the pressure antinodes of a standing wave and separate from blood components in under 60 seconds with >99% efficiency. fNACPs are subsequently fluorescently labeled in the pipette and are analyzed by both a custom, portable fluorimeter and flow cytometer. We demonstrate the detection of anti-ovalbumin antibodies from blood at picomolar levels (35 to 60 pM) with integrated controls showing minimal nonspecific adsorption. Overall, this system offers a simple and versatile approach for the rapid, sensitive, and specific capture of biomolecules.

## Teaser

An acoustic pipette and functional microparticle system is used for rapid and sensitive biomarker detection from whole blood.

## INTRODUCTION

Detection of biomolecules from biofluids using both qualitative and quantitative assays is essential for patient diagnosis and disease monitoring (*1*, *2*). Many qualitative assays benefit from user-friendliness, minimal points of error, and simple readouts that eliminate the need for complex instrumentation; however, they generally fail to enumerate biomarkers or detect rare (picomolar-level) analytes. For example, lateral flow assays (LFAs) are implemented widely for point-of-care (POC) testing due to their ease of use and simple readouts that can be analyzed by eye (*3*, *4*). However, LFAs typically cannot yield quantitative results, which lessens their usefulness in evaluating immune states on the individual and population levels (*5*, *6*). Quantitative assays, however, have the capacity to provide numerical readouts that offer a more comprehensive understanding of patient conditions. Enzyme-linked immunosorbent assays (ELISAs) are considered the gold standard for quantitative biomarker detection due to their ability to provide high sensitivity and specificity; however, ELISAs entail numerous blocking, labeling, and washing steps that require substantial user engagement for several hours (*7*, *8*). As a result, this assay and others like it introduce multiple potential points of user-related error that can lead to false results; moreover, they require the use of complex, expensive, and bulky instrumentation that limits their use in laboratory settings.

**Fig. 1.**
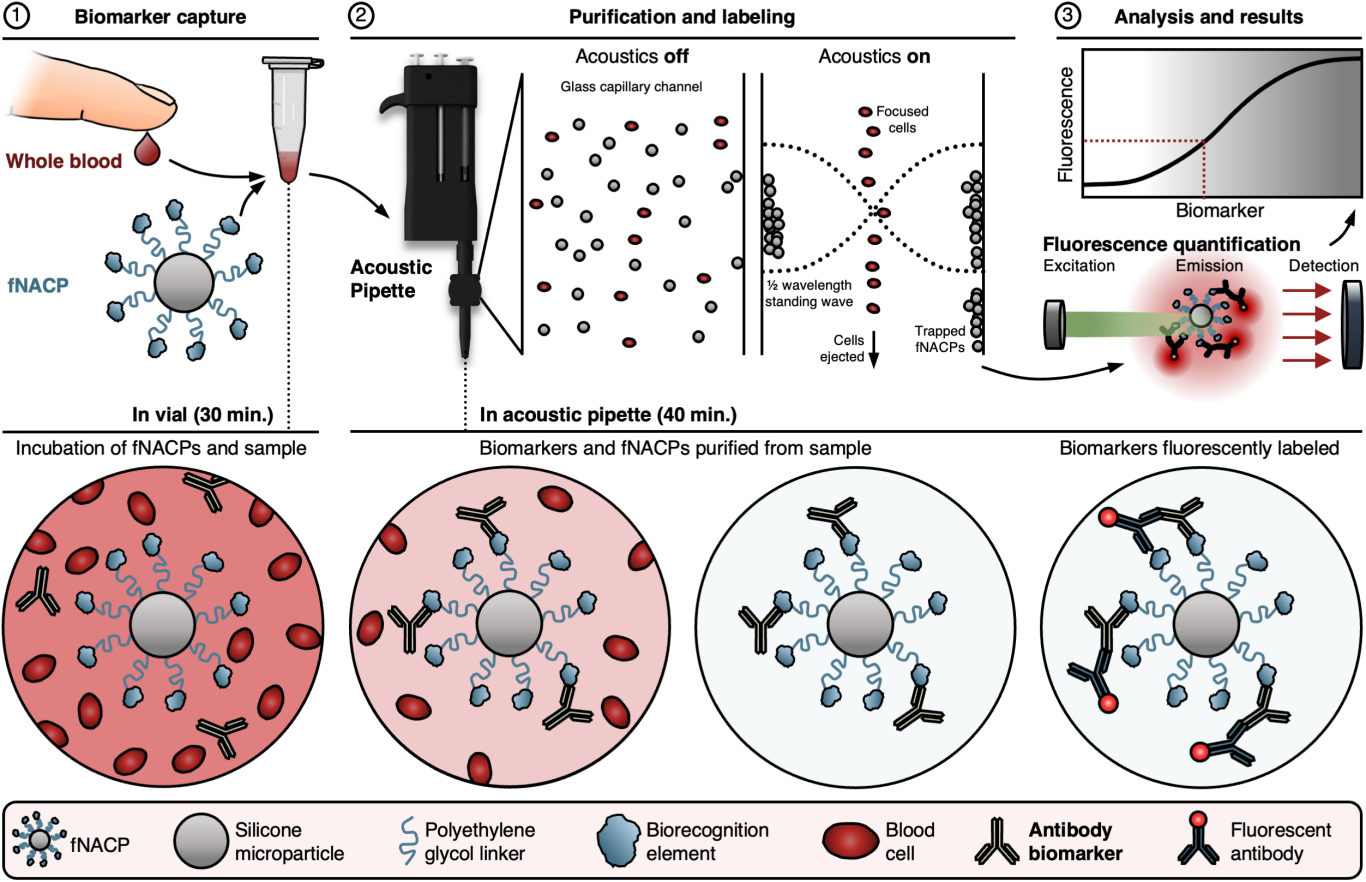
Schematic overview of the particle-based, acoustic pipette–enabled biosensing assay. fNACPs capture target biomarkers from whole blood samples. fNACPs are then purified from blood components by acoustic trapping, and captured biomarkers are labeled with a fluorescent antibody within the acoustic pipette. fNACP fluorescence is then measured to determine biomarker presence and concentration. Illustrations are not to scale.

To merge the benefits of qualitative and quantitative biosensing assays, we have developed a class of acoustically responsive functionalized microparticles and a handheld, ergonomic acoustic pipette for the rapid, sensitive, and user-friendly detection of biomarkers by downstream fluorescence analysis (Fig. 1, upper row). The presented system offers multiple advantages over ELISA while maintaining the same effective sensitivity. To accomplish this, the pipette contains an acoustofluidic trapping channel wherein biospecific functional negative acoustic contrast particles (fNACPs) are rapidly purified from whole blood via acoustofluidic trapping. Because of their negative acoustic contrast, fNACPs trap along the acoustofluidic channel walls while cells are pushed to the center of the channel due to their positive acoustic contrast. By relying on acoustic trapping, this technology (i) eliminates the need for extensive sample preparation, (ii) enables a simplified assay workflow with reduced points of potential user error, and (iii) decreases the overall assay time to only 70 min.

The fNACP functionalization approach we use allows for the simple tuning of fNACP biomarker specificity. To facilitate biomarker detection in this study, fNACPs were functionalized with antifouling polymer brushes terminated with antigen recognition elements to capture antibody biomarkers (Fig. 1, lower row). Using ovalbumin (OVA)–functionalized fNACPs, we show the sensitive detection of anti-OVA immunoglobulin G (IgG) antibodies at picomolar levels (35 pM) when analyzed by flow cytometry. By integrating a wash buffer and fluorescent secondary antibodies into the acoustic pipette, we demonstrate a simplified assay workflow in which, after capture of anti-OVA from whole blood, fNACPs can be rapidly purified from blood, washed, labeled with a fluorescent antibody, and ejected for fluorescence analysis with only ~10 min of user involvement. Enumeration of biomarker concentration can then be completed by comparison to fNACP-based assay calibration curves. Last, we show toward POC detection of anti-OVA IgG at 100 nM using a custom benchtop fluorimeter designed to house the acoustic pipette. The fluorimeter markedly simplifies fluorescence analysis compared to that which is required by flow cytometry, which in turn substantially broadens the range of situations in which the presented NACP-based assay may be applied. This technology could thus be useful for monitoring the health of patients or informing diagnoses for a breadth of diseases and conditions, such as viral infections, bacterial infections, cancer, and allergic reactions, which are detectible via measurement of biomarker levels in biofluids.

## RESULTS

### Production of trappable, low-polydispersity microparticles

Acoustic standing wave–based acoustofluidic separation leverages acoustic radiation forces to direct particles, cells, or other objects toward different stable positions along the standing wave. The primary acoustic force acting axially (i.e., perpendicular to the channel walls) on particles in an acoustic standing wave is given by

Fp=−(πp2Vpβf2λ) φ (β,ρ) sin (2 kx) (1)

where *p* is the pressure amplitude, *V*_p_ is the particle volume, λ is the ultrasonic wavelength, *x* is the distance from the pressure node, and *k* is defined as $\frac{2\pi }{\lambda }$. The acoustic contrast factor, φ(β, ρ), is given by

$$\varphi \;\left(\beta ,\rho \right)=\frac{5\rho_{p}-2\rho_{f}}{2\rho_{p}+\rho_{f}}-\frac{\beta_{p}}{\beta_{f}}$$ (2)

where variables ρ and β represent the density and compressibility, respectively, and the subscripts p and f denote the suspended object and the carrier fluid, respectively (*9*–*11*). When a half-wavelength standing wave is established within a microfluidic channel, such as a glass capillary, positive acoustic contrast particles (PACPs) with diameters much smaller than the wavelength (e.g., cells) move toward the pressure node, which is located along the middle of the channel (*12*). Alternatively, NACPs undergo acoustophoresis to the antinodes along the walls of the channel. In addition to the primary radiation force, particles and cells in the channel also experience a secondary radiation force, which is attractive between particles at close distances (*11*, *13*). Therefore, when NACPs undergo acoustophoresis, they are forced to the channel walls and subsequently aggregate. Last, beyond the axial component of the acoustic radiation force, particles in an acoustofluidic channel experience a lateral acoustic radiation force that originates from gradients in the local acoustic potential along the channel and acts parallel to the channel, toward regions of local acoustic minima or maxima. Under certain conditions, such as at high acoustic radiation forces, this can result in acoustic trapping, which we define as the immobilization of NACPs along the pressure antinodes in the presence of an applied flow, due to both the lateral component of the primary acoustic radiation force and the secondary acoustic radiation force exceeding the force on NACPs from Stokes’ drag (*13*, *14*). Previous works have shown use of continuous acoustic separation of NACPs from cells or other PACPs by an acoustic standing wave within trifurcating microfluidic channels; however, to our knowledge, static trapping of NACPs under continuous flow has not been rigorously demonstrated (*9*, *15*–*17*). This trapping approach eliminates the need for complex fluidic channels, relative to continuous separation, simplifying device manufacturing and reducing costs. Unlike trapping of NACPs, trapping of PACPs has been shown previously; however, because the fluid flow rate at the channel walls is much lower than that at the middle of the channel, trapping of NACPs can occur at throughputs much higher than trapping associated with PACPs (*14*, *18*–*20*). Last, because of the discriminant forces on cells and elastomeric particles in an acoustic standing wave, acoustofluidic technologies have the potential for higher separation purities than methods using optical, electrical, or magnetic fields (*21*, *22*).

**Fig. 2.**
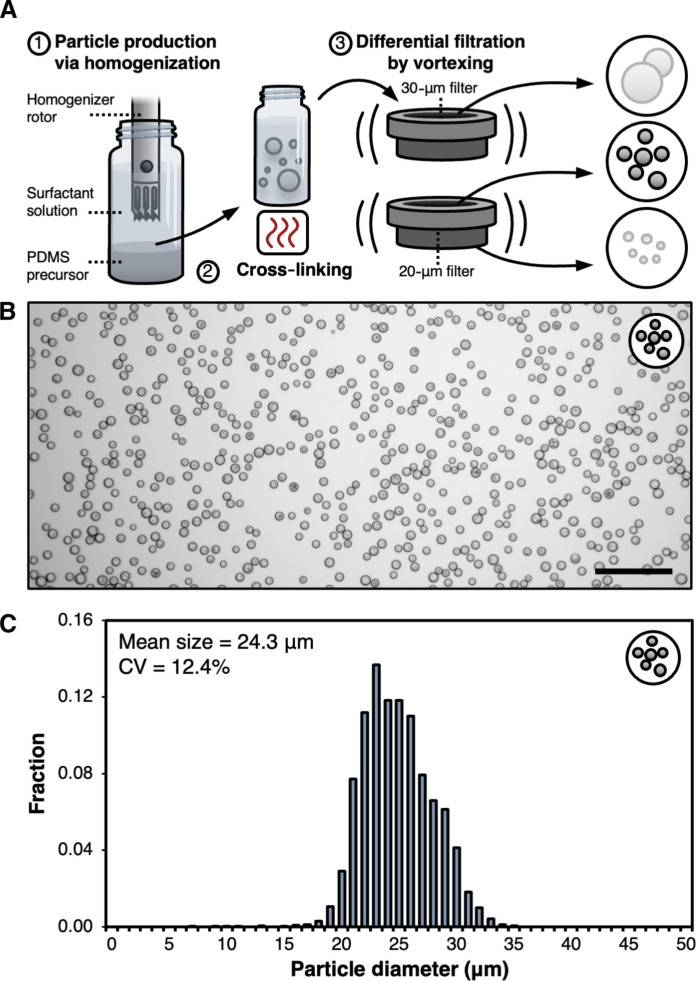
NACP production. (**A**) Schematic overview of NACP production. A PDMS precursor is homogenized to form droplets. The solution is heated to facilitate PDMS cross-linking. Cured particles are filtered while vortexing to isolate a low-polydispersity fraction of NACPs. (**B**) Bright-field microscopy image of isolated NACPs. Scale bar, 100 μm. (**C**) Histogram of isolated NACP sizes, as determined by image analysis (*N* = 3812).

To leverage the multiple benefits of separation via trapping of NACPs, we developed a technique to produce highly trappable, microscale NACPs. Base NACPs were first produced by homogenization of a polymer precursor containing 0.1% v/w triethoxyvinylsilane (TEOVS) in Sylgard 184 polydimethylsiloxane (PDMS) submerged in a solution of Pluronic F108 surfactant. PDMS was selected as the primary NACP material due to its inherent negative acoustic contrast (*10*), while TEOVS was included to yield functional silane groups on the surfaces of NACPs for later functionalization steps (*23*). After homogenization and formation of precursor droplets in an emulsion, the solution was heated to cure the PDMS and form polydisperse NACPs. As the use of polydisperse NACPs in a fluorescence-based assay was expected to increase variability of measurements (fig. S1), we opted to isolate a low-polydispersity fraction of the NACPs by sequential vortexed filtration through 40-, 30-, and 20-μm filters, collecting the NACPs retained by the 20-μm filter (Fig. 2A). This process yields NACPs with a low polydispersity (Fig. 2B) with a mean size of 24.3 μm [coefficient of variance (CV) = 12.4%; Fig. 2C]. These particles are large relative to many particles used in diagnostic assays (*24*). While the use of smaller particles in assays may offer the potential for faster particle-biomarker binding interactions due to increased diffusivity, we expected that the larger particle size also facilitates faster, more robust trapping of particles under flow as the magnitude of acoustic radiation forces scales proportionally with the volume of particles (cubically with the radius), as seen in Eq. 1 (*11*). As such, we anticipated that the use of larger, more trappable particles would enhance the isolation and recovery of captured biomarker. If desired, the NACP size could be easily tuned without modification of the production approach by simply selecting filters sized for the isolation of a smaller population of NACPs; however, a detailed investigation of the interplay between the particle size and total recovered biomarker is reserved for future studies.

**Fig. 3.**
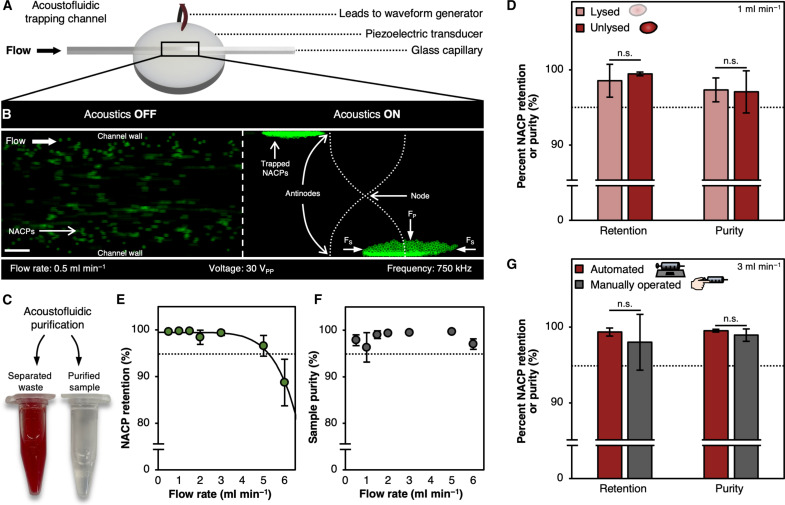
NACP trapping and purification from whole blood. (**A**) Schematic illustration of the acoustic trapping channel including the piezoelectric transducer, glass capillary, and electrical leads. (**B**) Acoustic trapping of NACPs within a glass capillary supporting a half-wavelength standing wave. NACPs, shown in green, were coated with FSA. Scale bar, 200 μm. (**C**) Representative images of separated waste, rich in cells, and a purified sample, rich in NACPs, after processing 5 × 10^4^ NACPs in 100 μl of unlysed blood diluted to 1 ml with the wash buffer in the acoustofluidic trapping channel at 0.5 ml min^−1^. (**D**) Comparison of NACP retention and sample purity after separation from lysed and unlysed blood samples, *N* ≥ 4. (**E**) NACP retention over a range of flow rates through the acoustofluidic trapping channel, *N* ≥ 4. (**F**) Sample purity over a range of flow rates through the acoustofluidic trapping channel, *N* ≥ 4. (**G**) Comparison of NACP retention and sample purity after separation by an automated syringe pump (red bars; *N* = 5) and manual operation (gray bars; *N* = 24). Data for manual operation are compiled from five individual operators, each with four or five individual samples. All data are presented as means ± SD, and significance between conditions was evaluated using one-tailed Student’s *t* tests. n.s., not significant.

To assess the acoustic trapping of NACPs, we fabricated an acoustofluidic trapping channel by affixing a square glass capillary with a 1 mm x 1 mm inner width and height to a piezoelectric transducer having a resonance frequency of 750 kHz (Fig. 3A). The transducer was actuated by applying an ac sine wave via leads connected to a function generator and amplifier. By operating the transducer at or near 750 kHz, a half-wavelength standing wave was established in the capillary channel. We monitored transducer heating by measurement of the transducer temperature after prolonged (>5-min) application of 750-kHz sine waves of increasing voltages (from 1 to 50 V_pp_) and determined that application of a 30-V_pp_ sine wave did not cause heating above 37°C; as such, transducers were operated at 30 V_pp_ for the remainder of the studies. We passed a solution of fluorescent streptavidin (FSA)–coated NACPs in phosphate-buffered saline (PBS) through the device at a flow rate of 0.5 ml min^−1^ and applied a 750-kHz sine wave to the transducer. We found that the NACPs were strongly trapped along channel walls (Fig. 3B and movie S1). Trapping was readily achieved due to the large volume of the NACPs (>20 μm in diameter) compared to a past study (*25*).

### Purification of NACPs from whole blood

Whole blood is replete with red blood cells (RBCs), white blood cells (WBCs), and platelets that have the potential to disrupt pressure waves by scattering, possibly reducing the ability to acoustically trap NACPs (*10*, *11*, *26*). Previously, researchers have either removed or lysed RBCs when conducting acoustofluidic separations from whole blood (*27*, *28*). However, use of an RBC lysis buffer requires an additional processing step, complicating and elongating assays. To evaluate the necessity of an RBC lysis step in the acoustic pipette, we mixed 5 × 10^4^ NACPs with 100 μl of whole porcine blood containing a sodium heparin anticoagulant, whereby WBCs were stained with the NucBlue Live Cell Stain and diluted to 1 ml with either a 1X RBC lysis buffer or wash buffer composed of 1% w/v bovine serum albumin (BSA) in PBS. We then passed the solutions through the acoustofluidic trapping channel at a flow rate of 0.5 ml min^−1^ with acoustics engaged and collected both the separated waste, rich in blood components, and the purified sample of NACPs (Fig. 3C). Using flow cytometry, we enumerated (i) NACP retention, quantified by dividing the number of NACPs in the purified sample by the total number of NACPs in the purified sample and waste, and (ii) sample purity, quantified by comparing the number of WBCs in the purified sample to the total number in both the purified sample and waste (fig. S2). We found that use of the RBC lysis buffer did not significantly improve NACP retention nor sample purity (Fig. 3D), indicating that RBC lysis is not necessary at the flow rate tested, simplifying assay workflows and increasing POC translatability.

Previous demonstrations of acoustofluidic separation of NACPs have been unable to achieve high flow rates, limiting their value in rapid diagnostics applications (*10*, *22*, *28*). The main challenge in increasing flow rates through acoustofluidic devices is that such increases result in NACP channel residence times that are insufficient for full separation. Li *et al.* (*9*) were able to achieve 97.5% NACP separation by continuous separation in a trifurcating channel, but the flow rate was limited to 50 μl min^−1^. We hypothesized that this limited throughput was a result of the smaller acoustic radiation forces on the NACPs due to their smaller sizes (i.e., 1.5 μm in the study by Li *et al.* compared to the 24.3-μm NACPs in this study). To evaluate the achievable throughput of our system, we again mixed 5 × 10^4^ NACPs with 100 μl of stained whole porcine blood and diluted to 1 ml with the wash buffer. We passed the mixtures through the device at 0.5 to 6.0 ml min^−1^ using a syringe pump and analyzed the resultant separated waste and purified samples as described earlier. We found that the retention of NACPs remained above 95% at flow rates up to 5 ml min^−1^, 100-fold higher than that achieved by Li *et al.* (Fig. 3E). Moreover, the sample purity remained above 95% for all evaluated flow rates (Fig. 3F). To ensure high (i.e., ~99%) NACP retention, all subsequent acoustofluidic purifications were performed at 3 ml min^−1^.

Characterization of the trapping channel described thus far used a syringe pump to establish precise flow rates. To assess manual operation of the acoustofluidic trapping channel, which is expected to lead to less precise flow rates, five human operators manually pushed the fluid through the device by a syringe while targeting a flow rate of 3 ml min^−1^. We found that manual operation of the device did not significantly differ from operation by a syringe pump, establishing that the trapping channel could be integrated into the handheld acoustic pipette without sacrificing separation performance (Fig. 3G and fig. S3).

### fNACP functionalization

**Fig. 4.**
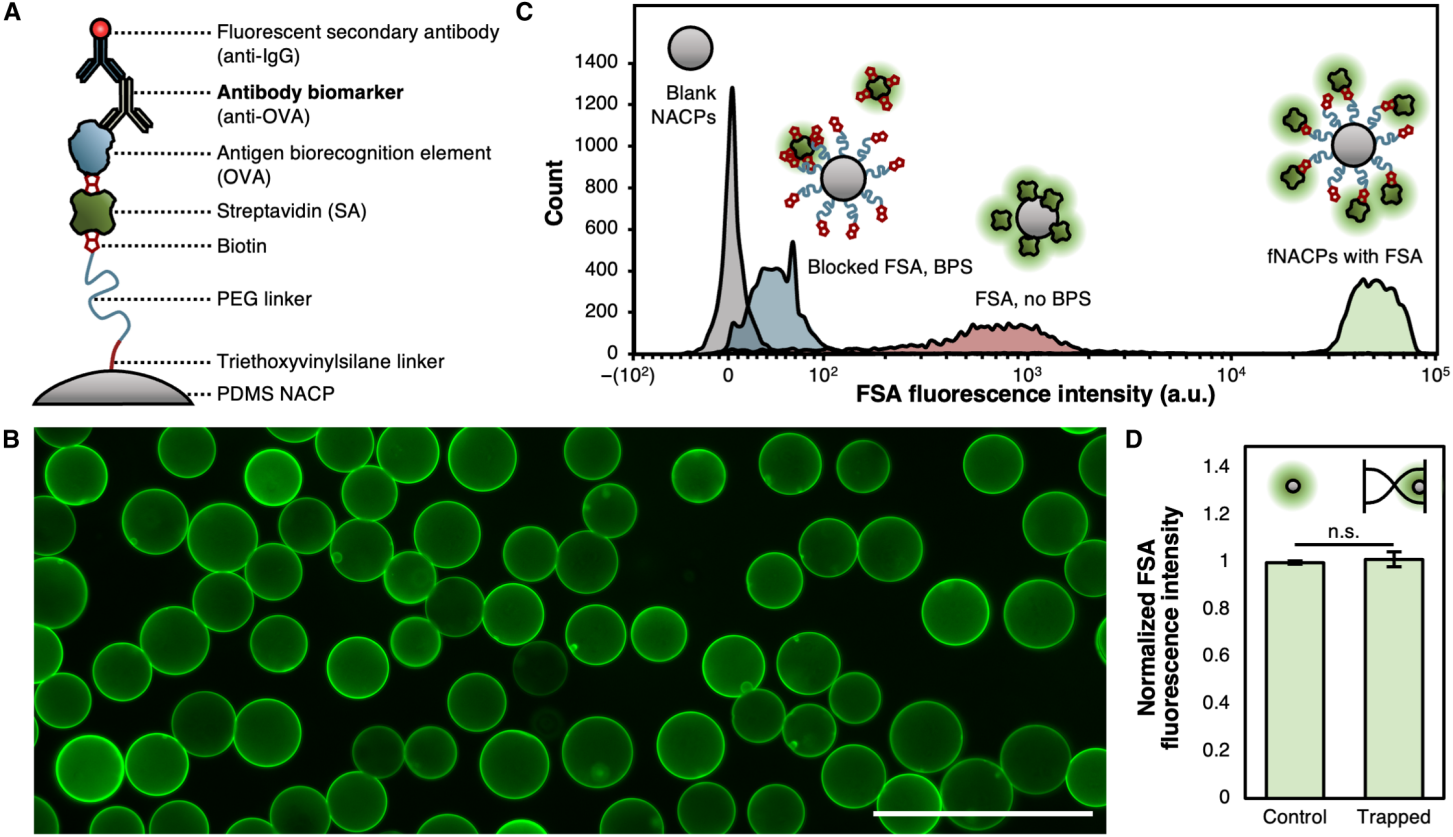
fNACP functionalization. (**A**) Schematic illustration of fNACP functionalization. (**B**) Fluorescence microscopy image of fNACPs functionalized with FSA. Scale bar, 100 μm. (**C**) Histograms of the fluorescence intensity of fNACPs without FSA, incubated with blocked FSA, incubated with FSA and without BPS, and with FSA and BPS as normal. Data are presented on a biexponential *x* axis. a.u., arbitrary units. (**D**) Comparison of NACP FSA fluorescence intensity with and without acoustic separation. Data are presented as median ± SD, *N* = 5. Significance between conditions was evaluated using a one-tailed Student’s *t* test.

To create a functional assay that leverages the NACPs, we designed an on-particle sandwich assay resembling ELISA, wherein target biomarkers are specifically captured by recognition elements bound to the surfaces of functionalized NACPs (fNACPs) and subsequently labeled by fluorescent secondary antibodies for analysis (Figs. 1 and 4A). When designing a biomolecule detection assay, it is crucial to minimize matrix effects as they may interfere with the assay and lead to false results (*29*). We speculated that nonspecific adsorption of proteins or other molecules to the fNACPs could result in improper labeling by the secondary antibody; as such, we designed the fNACPs to include a polyethylene glycol (PEG)–based antifouling layer (*30*, *31*). Thus, to functionalize base-NACPs, we bound 2-kDa biotin-PEG-silane (BPS) to silane-reactive groups from the incorporated TEOVS on NACP surfaces, yielding biotin-functionalized NACPs with an intrinsic antifouling coating. Biotin is a small molecule that binds strongly to SA, a moderate-sized (~52.8-kDa) protein with four biotin binding sites (*32*). Because of its four binding sites, addition of SA to the fNACPs enables subsequent conjugation of a variety of biotinylated recognition elements (e.g., antibodies and aptamers). Here, we selected biotinylated OVA as a model biorecognition element and anti-OVA IgG as a model biomarker. While the model biorecognition element and antibody biomarker pair is not clinically relevant, the proteins are structurally and functionally similar to many proteins that are. Thus, functionalization of fNACPs with more clinically relevant biotinylated recognition elements [e.g., severe acute respiratory syndrome coronavirus 2 (SARS-CoV-2) spike protein] would likely produce similar assay performances when detecting their respective clinically relevant biomarkers (e.g., anti–SARS-CoV-2 spike protein antibodies). Furthermore, anti-OVA is not naturally found in porcine blood, allowing us to spike in known concentrations. Notably, the inclusion of the PEG linker, in addition to providing antifouling properties, is expected to allow more favorable orientations of OVA to enhance anti-OVA capture, relative to OVA bound directly to the NACP surface (*33*).

### fNACP functionalization specificity

To investigate specificity of NACP functionalization, we produced low-polydispersity NACPs and functionalized their surfaces with the BPS linker. We then incubated BPS-decorated NACPs with FSA, yielding FSA-functionalized NACPs (Fig. 4B). We assessed FSA fluorescence via flow cytometry; as controls, we analyzed the fluorescence of blank NACPs, NACPs incubated with FSA blocked by free biotin, and NACPs without the BPS linker and incubated in FSA (Fig. 4C). Compared to the control groups, BPS-decorated NACPs incubated with FSA had substantially greater fluorescence intensity, suggesting that SA binding to fNACPs is specific and enhanced by the BPS linker. Moreover, the decreased fluorescence of NACPs with BPS and incubated with blocked FSA, relative to NACPs without BPS and incubated with FSA, provides evidence that inclusion of BPS suppresses nonspecific adsorption of proteins.

### fNACP functionalization durability

To assess if particles functionalized with SA are affected by conditions that will be present in a typical acoustic pipette–enabled assay, we mixed 5 × 10^4^ FSA-functionalized NACPs in 1 ml of the wash buffer and subsequently separated the mixture in an acoustic trapping channel. We measured the median fluorescence of the purified NACPs by flow cytometry. As a control, we also measured the fluorescence of FSA-functionalized particles from the same batch that did not undergo additional manipulation after functionalization (Fig. 4D). There was no significant decrease in fluorescence of the separated particles, indicating that NACP functionalization to the point of SA was resilient against the assay conditions associated with acoustofluidic separation.

### fNACP-based assay

**Fig. 5.**
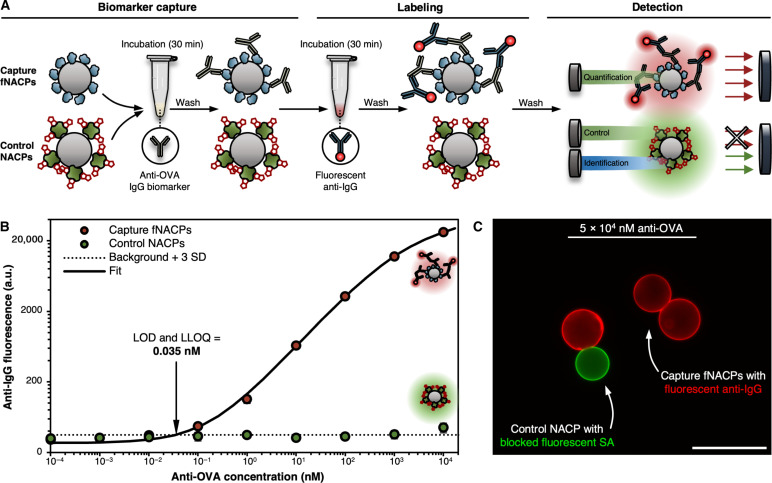
fNACP assay overview. (**A**) Schematic illustration of standalone NACP-based assay for anti-OVA detection. Capture fNACPs and control NACPs are incubated with anti-OVA, separated, and analyzed simultaneously by flow cytometry. (**B**) Capture fNACP and control NACP anti-IgG fluorescence over a range of anti-OVA concentrations spiked in buffer. Data were fit to a 5PL curve to determine the LOD and LLOQ. Data are presented as mean MFI ± SD, *N* = 3. Dotted line indicates the baseline fluorescence of fNACPs without fluorescent anti-IgG. (**C**) Overlaid fluorescence microscopy images of control NACPs and capture fNACPs after incubation with fluorescent anti-IgG after incubation with 5 × 10^4^ nM anti-OVA. Images were captured using green and red fluorescence channels. Scale bar, 50 μm.

To create an fNACP-based assay with a built-in control, we generated nonfluorescent anti-OVA–specific fNACPs (capture fNACPs) and fluorescent NACPs lacking a recognition element (control NACPs) (Fig. 5A). To generate the control NACPs, we functionalized NACPs with green FSA as described earlier and subsequently blocked remaining biotin-binding sites by incubation with a >12-fold molar excess of free biotin. During coincubation of capture fNACPs and control NACPs with samples containing spiked anti-OVA, anti-OVA should only bind to capture fNACPs. Then, during the subsequent labeling step, red fluorescent secondary antibodies should only bind to anti-OVA on the nonfluorescent capture fNACPs. During subsequent fluorescence analysis, the green fluorescent control NACPs can be checked for red fluorescence as an indicator of nonspecific adsorption (fig. S4).

### Standalone fNACP assay performance in buffer

We measured the performance of the fNACP-based assay by measuring the limit of detection (LOD), lower limit of quantification (LLOQ), upper limit of quantification (ULOQ), and dynamic range. We define the LOD in this study as the concentration of biomarker that yields an average median fluorescence intensity (MFI) 3 SDs above the median background fluorescence. The LLOQ and ULOQ are defined as the lowest and highest concentration of the biomarker, respectively, that can be reliably quantified, which is generally accepted to be that which yields a CV < 20%; the dynamic range is the range between the LLOQ and ULOQ (*34*, *35*). To quantify these metrics, we resuspended 1 × 10^4^ control NACPs and 1 × 10^4^ capture fNACPs in 100 μl of the wash buffer spiked with anti-OVA over the range of 10^−4^ to 10^4^ nM anti-OVA and incubated at room temperature for 30 min under agitation. The number of each NACP type used per sample (1 × 10^4^) was selected to ensure that sufficient numbers of NACPs were present for analysis by flow cytometry while also minimizing the total particle number to avoid potential dilution of signal across particles. The total surface area of the particles used was ~58% of that of a single well on a 96-well plate, commonly used in ELISA. After anti-OVA capture, the particles were incubated with fluorescent anti-IgG for 30 min. The particles were then washed and analyzed via flow cytometry (Fig. 5B and figs. S4 and S5). To enable enumeration of biomarkers and extract assay performance characteristics, experimental data were fit to a five-parameter logistic (5PL) curve, commonly used to fit experimental data from biosensing assays such as ELISA (*36*, *37*). Using this curve fit, the LOD and LLOQ were determined to be 0.035 nM, a sensitivity on the order of that reported for a commercial ELISA for anti-OVA (0.013 nM) (*38*). We were unable to determine an ULOQ as all investigated high anti-OVA concentrations yielded CVs < 20%. While the ULOQ could not be quantified, it is apparent that the dynamic range of the assay is much larger than that of commercial ELISAs (i.e., spanning at least six orders of magnitude, surpassing ELISA, which spans only ~2 orders of magnitude). The wide dynamic range of the assay relative to that of ELISA largely stems from the dissimilar signals and signal measurement approaches used in each assay. Fluorescence measurement systems typically allow measurement of fluorescence intensities at high cutoff values before signal saturation, whereas colorimetric measurements used for ELISA are limited by saturation of the optical density, restricting the dynamic range (*39*–*41*). As expected, the fluorescence of the control NACPs did not substantially increase with increasing anti-OVA concentration, indicating that the labeling by the secondary antibody was specific, as shown in a representative image of the control NACPs and capture fNACPs after completion of the assay (Fig. 5C).

### Acoustic pipette design and assay workflow

**Fig. 6.**
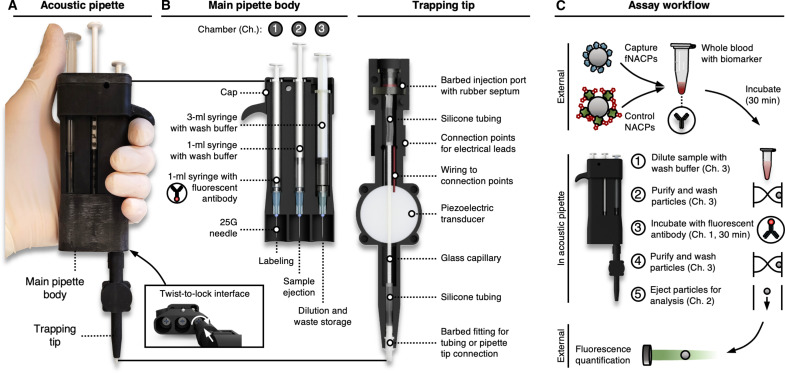
Acoustic pipette overview. (**A**) Image of the acoustic pipette. Inset shows the mechanism for movement and locking of the trapping tip. (**B**) Schematic illustration of acoustic pipette internals, including the main pipette body and trapping tip. (**C**) Schematic illustration of the general pipette-facilitated fNACP assay.

Using primarily 3D computer-aided design (CAD) and 3D printing, we created the ergonomic, handheld acoustic pipette (Fig. 6A and fig. S6). To accommodate all necessary assay components, the pipette was designed with two main parts: (i) the main pipette body, which contains three syringe chambers to store the wash buffer and fluorescent secondary antibody, and (ii) the trapping tip, which contains the acoustofluidic trapping channel and related components (Fig. 6B). The trapping tip is detachable from the main body of the pipette, and switching between chambers is facilitated by a facile twist-and-lock mechanism (Fig. 6, inset). The trapping channel is accessed by piercing an elastomeric injection port with needles attached to each of the three chambers in the main body of the pipette. Notably, the injection port prevents contamination between chambers and additionally prevents air from entering the channel when switching between chambers, which could cause disruption of the acoustic standing wave in the trapping channel. Pipette components are sized to process 100 μl of whole blood, which is a standard volume for many blood-based detection assays that use finger-prick sampling (*42*–*44*).

The pipette design enables a simple workflow that eliminates the many cumbersome steps associated with traditional immunoassays (Fig. 6C). Capture fNACPs and control NACPs are first resuspended in 100 μl of whole blood containing the target biomarker and are incubated outside of the pipette in a tube for 30 min. Then, the sample is diluted in the wash buffer from Chamber 3 of the pipette. The sample is then withdrawn from the sample tube with acoustics engaged, trapping the NACPs and fNACPs in the trapping tip, while the waste blood is pulled into Chamber 3 for containment. The particles are then further washed by pulling the fresh wash buffer through the channel and into Chamber 3. The trapping tip is then switched to Chamber 1, acoustics are disengaged, and the particles are pulled into Chamber 1 for incubation with fluorescent secondary antibodies for 30 min. After incubation, the particles are ejected back into the trapping tip with acoustics engaged. The particles are further washed by switching back to Chamber 3 and withdrawing the wash buffer past the particles. Last, the acoustics are disengaged and the wash buffer from Chamber 2 is used to eject the particles into tubes for analysis. Notably, user engagement is only required for ~10 min of the assay, and the entirety of the assay takes only ~70 min. Most of the assay time is consumed by the two 30-min incubation steps. Notably, this incubation time can vary based on the binding affinity between the recognition elements and the targets being detected as well as the concentrations of the various components. Optimization of these factors could potentially lead to even shorter assay times.

### Performance of acoustic pipette–assisted, fNACP-based assay in whole blood

**Fig. 7.**
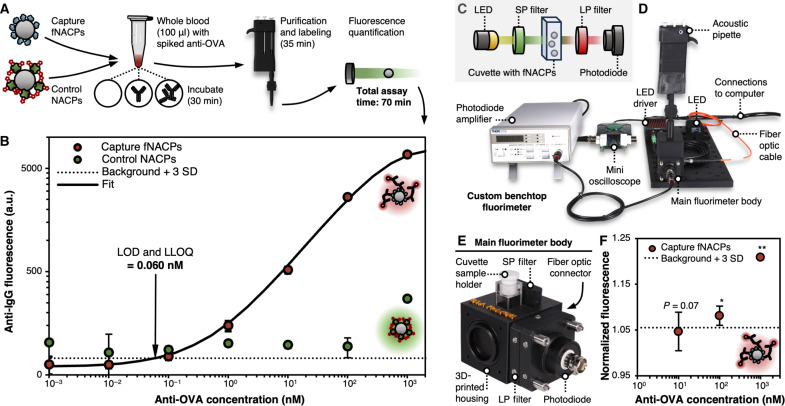
Detection of anti-OVA from whole blood in the acoustic pipette. (**A**) Schematic illustration of the workflow for detecting anti-OVA from whole blood in the acoustic pipette. (**B**) Fluorescence of anti-IgG on capture fNACPs and control NACPs over a range of anti-OVA concentrations spiked in whole blood. Data were fit to a 5PL curve to determine the assay LOD and LLOQ. Dotted line indicates baseline fluorescence of fNACPs without fluorescent anti-IgG. Data are presented as mean MFI ± SD, *N* = 3. (**C**) Schematic illustration of the primary components of the custom fluorimeter, including the LED source, an SP filter, a cuvette for containing the fNACP samples, an LP filter, and a photodiode detector. (**D**) Image of the custom benchtop fluorimeter and associated electronics. (**E**) Image of the main fluorimeter body, which houses all components from (C). (**F**) Capture fNACP anti-IgG fluorescence over a range of anti-OVA concentrations spiked in buffer, as measured by the custom fluorimeter. Data are presented as normalized signal ± SD. Significance between conditions was evaluated using a one-tailed Student’s *t* test. *N* = 3, *P < 5 × 10^−3^; **P < 5 × 10^−5^, relative to background.

To evaluate the fNACP-based assay after integration into the acoustic pipette, we incubated 1 × 10^4^ control NACPs and 1 × 10^4^ capture fNACPs in 100 μl of whole blood spiked with anti-OVA over the range of 10^−3^ to 10^3^ nM. After incubation, we used the acoustic pipette to purify, wash, and label the particles as described earlier (Fig. 7A). Upon analysis by flow cytometry, we found that the performance of the assay was similar to that of the standalone assay performed outside of the pipette in buffer, in terms of dynamic range, LOD, and general signal response. The measured LOD and LLOQ were both 0.06 nM; this value is minimally elevated relative to the standalone assay conducted in buffer, as can be expected when conducting an assay in a complex biofluid like whole blood (Fig. 7B). Again, the dynamic range was not fully defined, but no apparent saturation point was reached, indicating a dynamic range spanning at least five orders of magnitude. The control NACPs exhibited slightly elevated anti-IgG fluorescence at high anti-OVA concentrations, relative to the assay conducted outside the pipette. We speculate that this elevated fluorescence originated from nonspecific adsorption of proteins from blood, considering such elevation was not observed in the assay conducted in buffer alone. This suggests that detection of anti-OVA at high concentrations from whole blood may be inconclusive, necessitating retesting such samples after dilution. The control NACPs thus enable simple evaluation of assay performance and reliability, as designed. Future work dedicated to engineering the length, packing density, and composition of the antifouling polymer could further enhance the antifouling properties of the capture fNACPs and control NACPs to ultimately enhance assay performance in complex biofluids. Overall, the NACP-based assay performance remained predominantly unchanged when integrated with the acoustofluidic pipette and detecting biomarkers from whole blood samples.

### Design and performance of custom fluorimeter for fNACP-based assay analysis

While analysis of NACP fluorescence by flow cytometry helps to provide high sensitivity and a broad dynamic range owing to its use of narrow band laser excitation sources and highly sensitive photomultiplier tubes for emission detection, restricting analysis to flow cytometry is prohibitive to many POC testing situations due to bulkiness of—and lack of widespread access to—such instrumentation. To demonstrate integration of the NACP-based, acoustic-pipette enabled assay into a POC-compatible system, we designed a custom benchtop fluorimeter that can dock with the acoustic pipette and analyze fNACP fluorescence. By integrating the NACP-based assay, pipette, and fluorimeter, the resultant system is portable and capable of simplified completion of the entire assay, including capture, isolation, and detection of biomarkers. The main components of the fluorimeter include a light-emitting diode (LED) light source, a shortpass (SP) filter, a 100-μl cuvette, a longpass (LP) filter, and a photodiode (PD). During assays, fluorescent labels on the NACPs in the cuvette are excited by the filtered LED light, and light emission is detected by the photodiode (Fig. 7C and fig. S9). Notably, the 600-nm SP and 590-nm LP filters were selected specifically for analysis of the Alexa Fluor 594 fluorophore conjugated to the secondary antibody; by simply switching out these filters, other fluorophores can be detected by the system. Moreover, the cuvette size can be tuned depending on the volume of samples being tested, making the fluorimeter and its overall sensitivity customizable to application needs. The bulk of the setup, including a photodiode amplifier, mini oscilloscope, and the main fluorimeter body, easily fits on a benchtop. We designed and 3D printed a fluorimeter pipette docking cap to interface with the acoustic pipette; after all pipette-based assay processing has been completed, the pipette can be inserted directly into the fluorimeter to eject NACPs into the cuvette for analysis (Fig. 7, D and E). A more detailed description of the fluorimeter setup can be found in the Supplementary Materials.

While flow cytometry analyzes fluorescence of individual particles, the custom fluorimeter excites and detects light emission from a large cluster of particles simultaneously. This decreases measurement time and post-measurement analysis substantially, increasing ease of use as a result. To maximize the fluorescence signal from groups of particles, we modified the NACP-based assay by increasing the number of NACPs, as well as the sample volume, 10-fold. As the fluorimeter was designed for measurement of a single fluorophore, control NACPs were excluded from this study. Thus, the workflow for using this instrument is identical to that shown in Fig. 7A, with the only difference being the exclusion of control particles. To test the performance of the fluorimeter, we incubated 10^5^ OVA-functionalized fNACPs in 1 ml of the wash buffer spiked with 0, 10^1^, 10^2^, and 10^3^ nM anti-OVA, subsequently labeled the fNACPs with fluorescent secondary antibody, and measured the fluorescence using the fluorimeter. We measured significant detection at both 10^2^ and 10^3^ nM anti-OVA, as indicated both by statistical significance and by fluorescence signals at least 3 SDs above that of NACPs incubated with the secondary antibody and without anti-OVA (i.e., the background). While the signal at 10^1^ nM anti-OVA was not significantly different from that of the background sample, the calculated *P* value of 0.07 suggests that this concentration is near the detection limit of the described system and assay. Because the fluorimeter prototype uses a broadband LED excitation source and silicon photodiode for emission detection, rather than a laser excitation source and photomultiplier tube that are used in flow cytometers, this sensitivity is lower than that associated with flow cytometry; however, the modular design of the fluorimeter could be exploited to enhance sensitivity in future work. For example, the photodiode can be replaced with a more sensitive detector (e.g., avalanche photodiode) or a smaller cuvette could be used to further concentrate the NACPs. Overall, we have created the first prototype POC measurement instrument that can be integrated with the handheld acoustic pipette and fNACP-based assay to enable the rapid and simple isolation and detection of proteins without bulky, expensive instrumentation. These results highlight the flexibility and potential of the NACP-based assay to capture various different biomarkers from whole blood in a rapid and sensitive manner.

## DISCUSSION

We demonstrate an NACP-based, acoustic pipette–enabled assay that isolates biomarkers from whole blood for rapid and sensitive detection in a simple, ergonomic handheld device. This work integrates two key innovations: (i) fNACPs that specifically capture target biomolecules while suppressing nonspecific adsorption and (ii) an acoustic pipette that enables the rapid purification, washing, and labeling of biomarkers. We describe methods to synthesize large NACPs with a low polydispersity (i.e., <15% CV) that can be acoustically trapped and purified from whole blood with high efficiency at rates up to 5 ml min^−1^. We functionalized the NACPs to form fNACPs using a flexible conjugation approach that enables simple tuning of the fNACP specificity. We also designed a handheld acoustic pipette that contains all necessary components to conduct the fNACP-based assay, including the wash buffer, a fluorescent secondary antibody, and a trapping channel supporting a half-wavelength acoustic standing wave. Using the acoustic pipette in combination with flow cytometry, we developed a serological assay to enumerate captured anti-OVA in small volumes of whole blood by comparison with calibration curves. Because we use fluorescent labels and not enzymatic labels, such as those as used in traditional ELISA, the most comparable clinical technology for biomarker quantification, the calibration curves can be prepared ahead of sample analysis when using particles from the same batch and fluorescent antibodies from the same lot (i.e., with the same number of fluorophores per antibody). This results in rapid analysis of single samples. Using this assay, we detected anti-OVA from whole blood with an LOD of 0.06 nM, a sensitivity competitive with commercial ELISA kits. Beyond this, we developed a portable, custom benchtop fluorimeter for analysis of samples processed in the acoustic pipette. Using a modified fNACP-based assay, we rapidly detected anti-OVA at 100 nM. By integrating the pipette and fluorimeter, thereby eliminating the need for flow cytometry, we show the ability for the NACP-based, acoustics-enabled assay to be simplified for biomarker detection in areas with limited resources. Such measurements may be especially useful in situations where test results must be obtained in timeframes much shorter than that required for shipment to and analysis by external laboratories.

This platform technology has the potential to markedly shorten the time required to isolate and detect biomarkers (i.e., requiring only 70 min with less user engagement than commercial ELISA methods of 4 to 6 hours). By integrating the system with a low-cost benchtop fluorimeter, this technology could be of great use for POC testing where timely analysis of single samples is necessary. Furthermore, it enables facile adaptation for detecting a range of antibodies, antigens, toxins, nucleic acids, or other types of biomarkers through its simple and modular method of functionalizing fNACPs. While the presented fNACP-based assay only allows for detection of a single biomarker, by barcoding fNACPs displaying different biorecognition elements with varied amounts of fluorophore, this technology could enable simple, rapid, fNACP-based multiplexing to enhance diagnostic capabilities. Furthermore, while not currently suited for POC detection, with the addition of an on-board power supply or modification of the signal transduction approach, the integrated acoustic pipette and fluorimeter system holds potential to enable deployment of POC diagnostic testing in remote areas, while maintaining—and potentially increasing—assay sensitivity compared to mainstay detection techniques. The acoustic pipette and fNACPs can also be of use independent of one another; the acoustic pipette could be a powerful tool for the simple and rapid separation of a host of other particle or cell types in laboratories or elsewhere, while the NACPs may help to expand the capabilities of other acoustofluidic systems that currently primarily use PACPs for sensing, object manipulation, or other applications.

## MATERIALS AND METHODS

### NACP production

NACPs were produced by mixing Sylgard 184 base (Dow Chemical) with Sylgard 184 cross-linker (Dow Chemical) at a 10:1 ratio by weight to form the PDMS precursor. TEOVS (Sigma-Aldrich) was added to the precursor at 0.1% v/w, and the modified precursor was mixed thoroughly. Then, a surfactant solution composed of 1% w/w Pluronic F108 (Sigma-Aldrich) in deionized water (DIW) was added to the precursor at a 10:1 ratio of surfactant solution to precursor. The mixture was homogenized using a T10 basic homogenizer (IKA), producing an emulsion of PDMS precursor droplets in the surfactant solution. The mixture was cured for 2 hours at 80°C, yielding a polydisperse population of NACPs. To isolate a population of particles between 20 and 30 μm, these particles were then filtered through cell strainers (PluriStrainer) while vortexing at speeds sufficient to agitate liquid in the cell strainer. Specifically, the NACPs particles were first filtered with a 40-μm filter while vortexing to agitate the particles in the filter. Particles that passed through the filter were collected. The filter was washed with the Pluronic F108 solution occasionally to remove trapped particles. This process was then repeated with a 30-μm filter. The particles were then filtered with a 20-μm filter. Particles retained by the 20-μm filter were washed into a tube and collected. The last step was repeated once more to further reduce the polydispersity.

### NACP sizing

Filtered NACPs were pipetted onto a glass slide. Images of the microparticles were captured by a Zeiss AxioVert A1 TL/RL inverted fluorescence microscope equipped with an Axiocam 305 mono camera (Zeiss, Germany). NACP sizes were determined by analyzing captured images via the Fiji/ImageJ software.

### Functionalization of OVA-decorated fNACPs

Low-polydispersity NACPs were functionalized by conjugating the NACPs with a BPS linker and subsequently incubating BPS-decorated NACPs with SA. Typically, 5 × 10^6^ base NACPs were washed thrice with 0.004% v/v Tween 20 (Sigma-Aldrich) in DIW and resuspended in 50 μl of a solution (30 mg ml^−1^) of 2-kDa BPS (Laysan Bio) in PBS. Particles were allowed to incubate for 2 hours at room temperature while mixing on a VorTemp 56 shaking incubator (Labnet) at ~600 rpm with regular agitation by hand when particle settling was noted. NACPs were washed thrice with 0.004% v/v Tween 20 in PBS (Sigma-Aldrich). NACPs were resuspended in 100 μl of SA or Alexa Fluor 488–conjugated SA (1.7 mg ml^−1^; Thermo Fisher Scientific) in PBS and incubated for 90 min at room temperature while mixing. After incubation, NACPs were washed thrice with 0.004% v/v Tween 20 in PBS (Sigma-Aldrich) and resuspended in 200 μl of biotin-OVA (2.5 mg ml^−1^; Nanocs) in PBS. NACPs incubated for 90 min at room temperature while mixing. Last, the fully functionalized fNACPs were washed thrice in the wash buffer composed of 1% w/v BSA (Sigma-Aldrich) in PBS. After washing, the fNACPs were resuspended in the wash buffer and stored at ~4°C until use. All wash steps in this protocol and subsequent procedures involved centrifuging the solution at 3000*g* for 3 min, aspirating the supernatant, and resuspending the pellet in the same volume of fluid. All mixing and incubation steps described in this procedure and subsequent procedures were performed in 1.5-ml low protein binding microcentrifuge tubes (Thermo Fisher Scientific).

### Functionalization of control NACPs

NACPs were functionalized with FSA as described earlier. Then, ~2.5 × 10^6^ FSA-conjugated particles were incubated in 100 μl of free biotin (13.3 mg ml^−1^; VWR), representing >12-fold molar excess to FSA, in PBS for 90 min while mixing at room temperature. Then, control NACPs were washed thrice in the wash buffer. After washing, fNACPs were resuspended in the wash buffer and stored at ~4°C until use.

### Evaluation of fNACP functionalization specificity

For these studies, FSA in solution was preblocked with free biotin prior to incubating with fNACPs to evaluate capture specificity. Blocked FSA was produced by adding a ~12.7-fold molar excess of free biotin and incubating for 1 hour to yield a solution (1.7 mg ml^−1^) of biotin-blocked, Alexa Fluor 488–conjugated SA. NACPs were incubated with or without BPS as described earlier and subsequently incubated with FSA. The MFI of particles (i) without BPS or FSA, (ii) without BPS but with FSA, (iii) with BPS and blocked FSA, and (iv) with BPS and FSA was measured using a BD FACSCelesta flow cytometer (BD Biosciences).

### Evaluation of fNACP functionalization durability

NACPs were functionalized with FSA as described earlier, and 5 × 10^4^ particles were diluted to 1 ml in the wash buffer. Fluorescent NACPs were then trapped in a trapping channel in the dark at a flow rate of 1 ml min^−1^ with an applied signal of 30 V_pp_ and 741 kHz. While NACPs were trapped, an additional 1 ml of the wash buffer was pulled through the channel to remove any unbound FSA. After trapping, the acoustics were disengaged, NACPs were ejected from the channel, and their fluorescence was analyzed by flow cytometry. NACPs from the same functionalization batch that were not passed through the trapping channel were used as a control.

### Standalone fNACP assay

A total of 1 × 10^4^ control NACPs and 1 × 10^4^ capture fNACPs were resuspended in 100 μl of the wash buffer spiked with rabbit anti-chicken OVA (Bio-Rad) over the range of 10^−4^ to 10^4^ nM anti-OVA. Particles were incubated for 30 min at room temperature while mixing. After incubation, particles were washed thrice in the wash buffer and resuspended in a solution (10 μg ml^−1^) of Alexa Fluor Plus 594–conjugated donkey anti-rabbit IgG (Thermo Fisher Scientific) in PBS. Particles were again incubated at room temperature while shaking for 30 min. After incubation, we washed the particles and analyzed the MFI of the anti-IgG on single NACPs via flow cytometry. Control NACPs were identified by FSA fluorescence.

### Trapping channel fabrication and operation

The trapping channel was fabricated by attaching a ~50-mm-long, 1 mm x 1 mm inner width and height square glass capillary (VitroCom) to a thickness mode piezoelectric transducer with a resonance frequency of 750 kHz (STEMINC MD20T27F750S, *t* = 2.7 mm, *d* = 20 mm) using cyanoacrylate glue (Gorilla Glue Company). Twenty-four AWG wires (Digikey), used as transducer leads, were soldered to either side of the transducer. Silicone tubing (Freudenberg Medical) was then attached to either end of the capillary and sealed with cyanoacrylate glue and Parafilm (VWR). For experiments with the acoustic pipette, a barbed Luer adapter (Fisher Scientific) was then attached to one side of the tubing. A rubber septum was cut to size from a 0.8-mm-thick silicone rubber sheet (Grainger) and inserted into the Luer adapter. A 3D-printed needle guide was then inserted into the adapter to compress the septum, forming a sealed injection port. The final device was then placed into a 3D-printed trapping tip. The transducer leads were coupled with leads from a waveform generator (33210A, Agilent) and amplifier (75A250AM2, Amplifier Research) connected in series. The applied frequency and voltage were measured using an oscilloscope (InfiniiVision, Keysight). Trapping channels were operated by applying a sinusoidal wave at ~750 kHz and 30 V_pp_ based on a series of optimization studies (see the Supplementary Materials for details).

### Evaluation of NACP trapping

A total of 5 × 10^4^ NACPs were resuspended in 100 μl of blood (whole porcine blood with sodium heparin, Lampire Biological Laboratories). Lysed blood samples were produced by adding 100 μl of the 10x RBC lysis buffer (eBiosense RBC lysis buffer, Invitrogen) and 800 μl of the wash buffer in PBS. Unlysed blood samples were produced by adding 900 μl of the wash buffer. Two drops of the NucBlue Live Cell Stain (Thermo Fisher Scientific) were added to each sample to stain WBCs for subsequent analysis by flow cytometry. To prepare the trapping channel, 4 ml of DIW was passed through the device to clean it and to remove air. Then, a 3-ml syringe (BD Biosciences) was attached to a GenieTouch syringe pump (Kent Scientific). The leads of the trapping channel were attached to the waveform generator and oscilloscope, and the channel was attached to the 3-ml syringe by silicone tubing. Samples were mixed on a vortexer and then withdrawn through the device at a fixed flow rate (i.e., 0.5 to 6.0 ml min^−1^) with the acoustics engaged. After the 1-ml sample had been withdrawn, 0.5 ml of the wash buffer was withdrawn at the same flow rate with acoustics on to wash the trapped particles and remove any residual blood components. The syringe containing the 1.5 ml of waste was removed, ejected into a 1.5-ml microcentrifuge tube, and replaced with a 1-ml syringe (BD Biosciences) containing 800 μl of the wash buffer. The acoustics were deactivated, and the new wash buffer was injected into the device at 2 ml/min, washing out trapped particles into an empty 1.5-ml microcentrifuge tube. The device was rinsed with DIW or the wash buffer between each sample. Manual device operation experiments were performed identically, but the syringes were operated by hand rather than a syringe pump. Operators targeted a flow rate of 3 ml min^−1^ by ejecting a known volume of fluid in a set amount of time while attempting to maintain a steady flow. For experiments involving video or image captures during NACP trapping, the trapping channel was placed on an inverted microscope stage with the transducer oriented along top of the channel.

### Flow cytometry for trapping evaluation

All samples were analyzed for purity and retention using flow cytometry. The cytometer was operated for 90 s per sample. Waste samples were analyzed at a flow rate of 60 μl min^−1^, and purified samples were analyzed at 12 μl min^−1^. Gating involved isolation of NACPs based on forward and side scatter (see the Supplementary Materials for details), while gating of WBCs involved measurement of the fluorescence of the nuclear stain. NACP retention was defined as

$$\text{NACP retention}=\frac{{\text{NACP}}_{S}}{{\text{NACP}}_{S}+{\text{NACP}}_{W}}\times 100\%$$ (3)

where NACP_S_ denotes the number of NACPs remaining in the purified sample and NACP_W_ denotes the number of NACPs in the waste fraction. Sample purity was defined as

$$\text{Sample purity}=\left(1-\frac{{\text{WBC}}_{S}}{{\text{WBC}}_{S}+{\text{WBC}}_{W}}\right)\times 100\%$$ (4)

where WBC_S_ denotes the number of WBCs found in the purified sample and WBC_W_ denotes the number of WBCs found in the waste.

### Acoustic pipette design and fabrication

Most acoustic pipette components, including the main body and trapping tip, were designed in AutoCAD Fusion 360 and 3D printed on a masked sterolithography 3D printer (SL1S, Prusa Research) using Tough Black resin (Prusa Research). STL files of the main acoustic pipette components are available and described in the Supplementary Materials. Printed parts were washed in isopropyl alcohol (Thermo Fisher Scientific) and underwent post-print drying and curing within a curing chamber (CW1S, Prusa Research) before device assembly. Parts were designed to be assembled through pressure fitting. Chambers in the main body consisted of a single 3-ml syringe and two 1-ml syringes (BD Biosciences), and 25G needles (BD Biosciences) were used to pierce the injection port of the trapping tip. Pipette assembly schematics are available in the Supplementary Materials (figs. S7 and S8).

### Acoustic pipette–enabled assay

The acoustic pipette was typically prepared by filling Chamber 1 with 100 μl of a solution (40 μg ml^−1^) of Alexa Fluor Plus 594–conjugated donkey anti-rabbit IgG, Chamber 2 with 400 μl of the wash buffer, and Chamber 3 with 1 ml of the wash buffer. Capture fNACPs and control NACPs were resuspended in 100 μl of whole porcine blood with spiked anti-OVA spanning 10^−3^ to 10^3^ nM. Samples incubated outside of the pipette in 1.5-ml microcentrifuge tubes for 30 min while mixing at 1200 rpm on a VorTemp mixer. Samples were then diluted to 1 mL with the wash buffer from Chamber 3 of the pipette. Samples were then withdrawn from the sample tube with acoustics engaged, trapping the particles in the channel, while the waste blood was pulled into Chamber 3 for containment. The particles were then further washed by pulling 500 μl of the fresh wash buffer through the channel and into Chamber 3. The trapping tip was then switched to Chamber 1, the acoustics were disengaged, and the particles were withdrawn into Chamber 1 to be labeled by the fluorescent secondary antibody. Particles were then incubated for 30 min in Chamber 1. After incubation, the particles were passed back into the trapping tip with acoustics engaged. Particles were further washed by switching back to Chamber 3 and withdrawing 500 μl of the fresh wash buffer past the trapped particles. Last, the trapping tip was switched to Chamber 2, the acoustics were disengaged, and the particles were ejected into sample tubes for analysis by flow cytometry. To generate a concentration response curve, this procedure was repeated for each concentration of anti-OVA in spiked in whole blood. After each sample was processed, NACPs were removed from the device and placed on a rotator during incubation for 36 min to allow time for the concurrent processing of multiple samples. After all samples were processed, samples were inspected by flow cytometry as described earlier.

### Custom fluorimeter design, assembly, and use

The custom fluorimeter consisted of the main fluorimeter housing, filtering optics, photodetector, photodiode amplifier, LED driver, LED, data acquisition unit, and computer. The main housing blocked the external light and held the cuvette sample, aligning it to the incoming LED light. A pipette docking cap sat on top of the housing and allowed the pipette to mate with the fluorometer housing and inject a sample into the cuvette below. The main fluorimeter housing, pipette docking cap, SP and LP filter holders, and photodiode holder were designed using AutoCAD Fusion 360 and 3D printed in matte black PLA using a Bambu Labs P1S 3D printer. STL files of the main housing and associated components are available and described in more detail in the Supplementary Materials. A commercially available LED driver (Thorlabs) set to 700 mA drove a high-power 9.9 mW, 565-nm fiber-coupled LED. Emitted LED light traveled through a 1-m-long and 400-μm in diameter multimode SMA-SMA fiber patch cable (Thorlabs) to a threaded SMA to SM1 fiber adapter (Thorlabs) with a 20-mm focal length collimating lens, mounted in one of the four 25.4-mm housing side ports. Light was filtered through a 600-nm SP filter (Thorlabs) before entering the cuvette chamber. Collimated light striking the Alexa Fluor Plus 594 fluorophore on fNACPs inside the cuvette caused fluorescence emission with a peak at 617 nm. Photons emitted 90° relative to the incident light passed through a 590-nm LP filter with a 610-nm pass wavelength (Edmund Optics) before being received by a silicon photodiode with 350- to 1100-nm sensitivity (Thorlabs). Photocurrent produced by the silicon photodiode was measured by a benchtop photodiode amplifier (Thorlabs), producing a ±10-V analog output with a gain *A*_PD_ of 10^8^ ± 5%. Notably, this photodiode amplifier can be replaced by a custom credit card–sized electronics board to further enhance portability of the fluorimeter in future iterations. Analog output was acquired with a portable USB oscilloscope (Digilent), and the waveform was recorded on a laptop to a CSV file using Waveforms (Digilent) at a rate of 100 Hz. Resulting signal characteristics and statistics were processed using Microsoft Excel. Additional details regarding specific components, part numbers, and function of the custom fluorimeter can be found in the Supplementary Materials.

### Custom fluorimeter-enabled assay

A total of 1 × 10^5^ capture fNACPs functionalized with OVA were resuspended in 1000 μl of the wash buffer spiked with rabbit anti-chicken OVA (Bio-Rad) over the range of 10^1^ to 10^3^ nM anti-OVA. Particles were incubated for 30 min at room temperature while mixing. After incubation, particles were washed thrice in the wash buffer and resuspended in a solution (10 μg ml^−1^) of Alexa Fluor Plus 594–conjugated donkey anti-rabbit IgG (Thermo Fisher Scientific) in PBS. Particles were again incubated at room temperature while shaking for 30 min. After incubation, particles were washed, resuspended in 100 μl of the wash buffer, transferred to the fluorimeter cuvette, and lastly analyzed using the custom fluorimeter. Fluorescence measurements were taken immediately after transferring the fNACPs to the cuvette to ensure that particles were suspended during measurements.

### 5PL fitting

Concentration response curves, shown in Figs. 5B and 7B, were fit with a 5PL curve to determine the LOD. To do so, we fit experimental data to the standard 5PL equation, given by

$$y=b+\frac{a-b}{{\left[1+{\left(\frac{x}{c}\right)}^{d}\right]}^{g}}$$ (5)

where *y* is the fluorescence intensity, *x* is the anti-OVA concentration, *c* is the mid-range concentration, *d* is the slope factor, *a* and *b* are the fluorescence intensities at the minimum and maximum anti-OVA concentrations, respectively, and *g* is the asymmetry factor. MATLAB was used for all fitting. The LOD was determined by identifying the concentration at which the response was 3 SDs above the background response (i.e., NACPs without any fluorescent labels), as predicted by the curve fit. To confirm that the fits were acceptable, we calculated the percent relative error of experimentally measured values relative to those predicted by the fits and ensured that the average relative error at each concentration fell below 25%. This data, along with fitted parameters for both standard curves shown in Figs. 5B and 7B, can be found in fig. S10 and table S1 of the Supplementary Materials.

### Statistical analysis

Statistical analyses were performed using Microsoft Excel. Differences between groups were compared by one-tailed Student’s *t* tests, and *P* < 0.05 was considered significant in the analyses. All quantitative data are expressed as the means ± SD.

## Supplementary Materials

### The PDF file includes:

Additional methods, results, and discussion Figs. S1 to S10 Tables S1 to S9 Legend for movie S1 Legends for stl files S1 to S11

### Other Supplementary Material for this manuscript includes the following:

Movie S1

STL files S1 to S11
