# Supplementary material for: Acoustic pipette and biofunctional elastomeric microparticle system for rapid picomolar-level biomolecule detection in whole blood
Source: Sci Adv. 2024 Oct 16;10(42):eado9018. doi: 10.1126/sciadv.ado9018 (PMC11482303; doi:10.1126/sciadv.ado9018)
Supplement: Supplementary file 1 — Additional methods, results, and discussion Figs. S1 to S10 Tables S1 to S9 Legend for movie S1 Legends for stl files S1 to S11 [file sciadv.ado9018_sm.pdf]

Science Advances
AAAS

Supplementary Materials for

Acoustic pipette and biofunctional elastomeric microparticle system for rapid picomolar-level biomolecule detection in whole blood

Cooper P. Thome *et al.*

Corresponding author: C. Wyatt Shields IV, charles.shields@colorado.edu

Sci. Adv. **10**, eado9018 (2024)
DOI: 10.1126/sciadv.ado9018

The PDF file includes:

Additional methods, results, and discussion
Figs. S1 to S10
Tables S1 to S9
Legend for movie S1
Legends for stl files S1 to S11

Other Supplementary Material for this manuscript includes the following:

Movie S1
STL files S1 to S11

Additional methods, results, and discussion.

Optimization of the acoustic waveform. Before use in experiments, frequency sweeps from 730–770 kHz were conducted on individual trapping channels to determine the proper operating frequency, which was estimated based on the extent of heating and the magnitude of the voltage across the transducer at a given frequency, with minimal heating and maximum voltage being targeted. Transducers were also assessed for heating during operation at 5–40 V_{pp}, and 30 V_{pp} was selected as the standard applied voltage as transducer temperatures did not exceed 37˚C with continuous operation (i.e., >10 min) at this voltage.

NACPs of reduced polydispersity for fNACP-based assays. As described in the main text, a low polydispersity fraction of NACPs was isolated from the polydisperse bulk of NACPs produced by homogenization. The reasoning for this was twofold: first, polydisperse NACPs, such as those filtered without vortexing to remove NACPs larger than ~50 µm, appear as a large population on forward scatter (FSC) versus side scatter (SSC) flow cytometry plots (Fig. S1A). This population overlaps with the typical location of blood components on the same plot, preventing the ability to differentiate NACPs from blood components based on FSC and SSC alone, as can be done with the NACPs that were vortexed while filtering to reduce retention of NACPs smaller than the filter size (Fig. S1B). Second, due to the wide distribution of particle sizes in the NACPs filtered without agitation, the fluorescence intensity distribution of the NACP population is sufficiently broad to reduce confidence for anti-OVA detection assays (Fig. S1C). After filtration with vortexing, the fluorescence intensity distribution of the NACPs is significantly narrower (Fig. S1D).

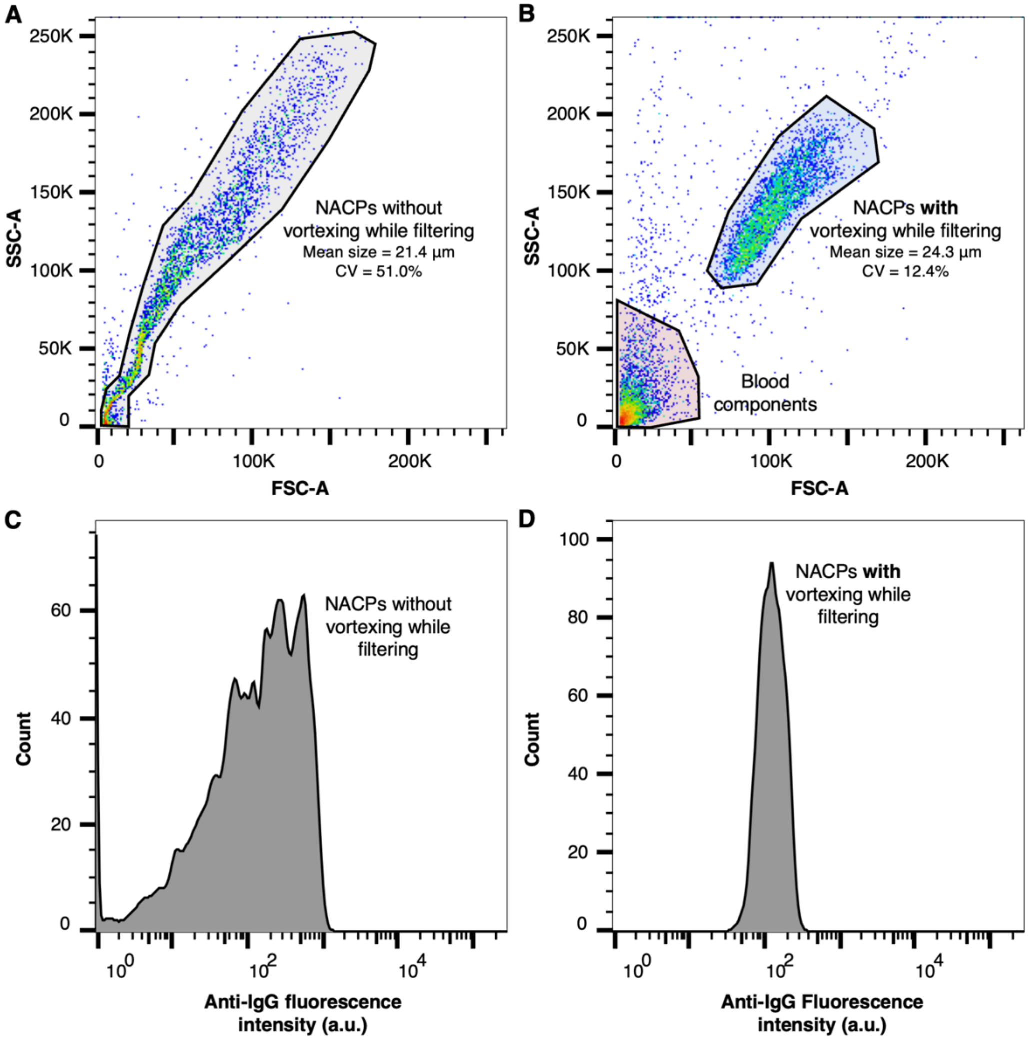

Fig. S1. Flow cytometry of unfiltered and filtered NACPs. (A) Representative FSC vs. SSC plot of NACPs that were filtered without vortexing. **(B)** Representative FSC vs. SSC plot of NACPs filtered as described in the main text and mixed with whole blood. **(C)** Anti-IgG fluorescence intensity of the NACPs from (A) not incubated with fluorescent anti-IgG. **(D)** Anti-IgG fluorescence intensity of the filtered NACPs from (B) not incubated with fluorescent anti-IgG.

Flow cytometry gating for NACP trapping evaluation. During analysis of separated waste and purified samples, as described in the text, blood components and NACPs were gated based on FSC and SSC (Fig. S2A,D). Within the blood component gate, WBCs were readily identified based on a comparison of two fluorescence channels, 450 nm emission with a bandwidth of ±40 nm and 535 nm emission with a bandwidth of ±50 nm, both with excitation by a 405 nm laser (Fig. S2B).

By gating in this manner, NACPs and WBCs could be isolated from RBCs and other debris (Fig. S2C).

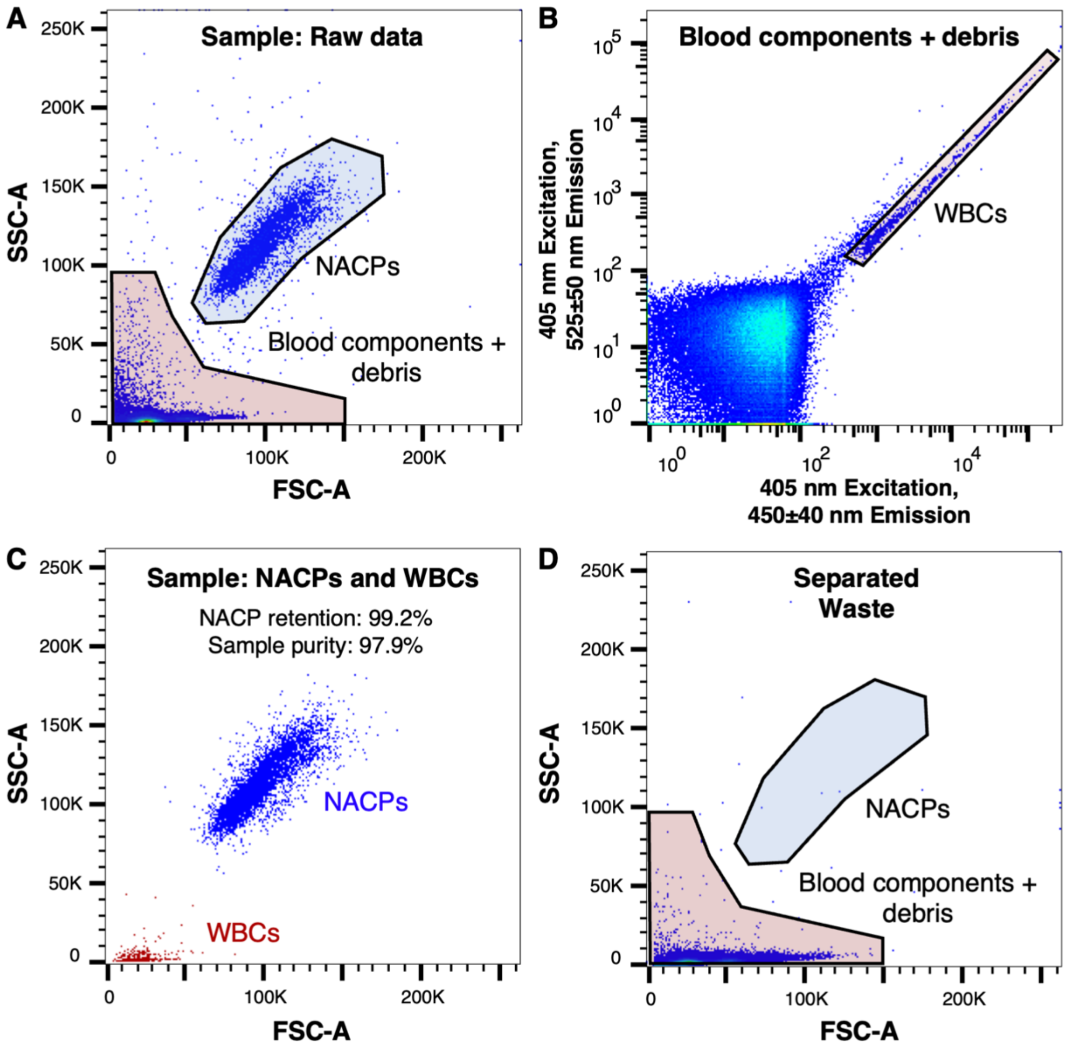

Fig. S2. Representative plots of flow cytometry gating for NACP trapping evaluation. (A) Representative FSC vs. SSC plot of a raw data from a purified sample from trapping experiments with gates showing the NACPs and the blood components. **(B)** Representative plot showing the gating of WBCs in the purified sample stained by NucBlue. **(C)** Representative FSC vs. SSC plot of NACPs and WBCs gated in (A) and (B), including calculated NACP retention and sample purity. **(D)** Representative FSC vs. SSC plot of the waste from trapping experiments with gates showing the NACPs and the blood components. Measurements in (D) are used to calculate NACP retention and sample purity in (C).

Extended manual trapping operation data. Individual results for NACP retention and sample purity from the manual trapping study, shown in cumulative form in Fig. 3G of the main text, are

shown in Fig. S3. NACP retention and sample purity did not differ significantly between any of the operators.

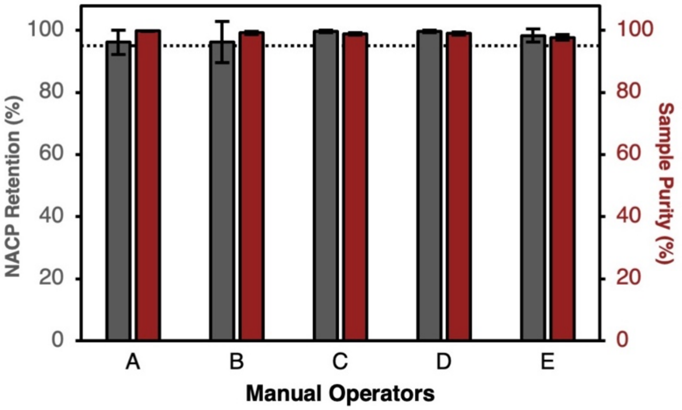

Fig. S3. Individual operator results from manual trapping study. Data is presented as mean ± standard deviation, N = 5 (A-D) or 4 (E). Dotted line shows 95% target NACP retention and sample purity.

Flow cytometry gating for fNACP-based assay. Control NACPs and capture fNACPs were gated by FSC and SSC (Fig. S4A). Within this population, control NACPs were differentiated by increased fluorescence from FSA that was exclusively used on control NACPs (Fig. S4B). Both populations were also analyzed for anti-IgG fluorescence, with the median anti-IgG fluorescence intensity of the fNACP population being correlated to anti-OVA concentration (Fig. S4C).

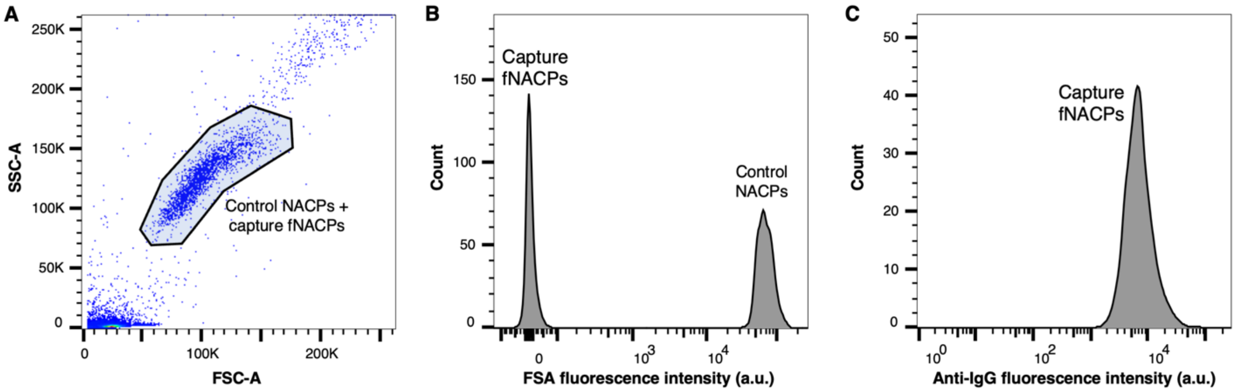

Fig. S4. Representative plots of flow cytometry gating for fNACP-based assay. (A) Representative FSC vs. SSC plot of a purified sample from the acoustic pipette with gates showing the NACPs and blood components. **(B)** Representative plot of FSA fluorescence intensity of the gated control NACPs and capture fNACPs from (A). (**C)** Representative plot of anti-IgG fluorescence on the capture fNACPs identified from (B).

fNACP-based detection specificity. To assess fNACP specificity, we incubated the fNACPs in buffer with no anti-OVA or 0.1 nM anti-OVA for 30 min, followed by incubation with fluorescent anti-IgG for 30 min. We found that the median anti-IgG fluorescence of control fNACPs and fNACPs incubated with only fluorescent anti-IgG were not statistically different, while the fNACPs incubated with fluorescent anti-IgG after anti-OVA showed dramatically increased fluorescence (Fig. S5). These results provided two key insights: first, labeling of fNACPs by the secondary antibody is specific, and thus, the system has a high signal-to-noise ratio, which is crucial for achieving high sensitivity assays. Second, because nonspecific adsorption of the secondary antibody was negligible for the group lacking anti-OVA, the results provide further evidence that fNACPs are resistant to nonspecific adsorption.

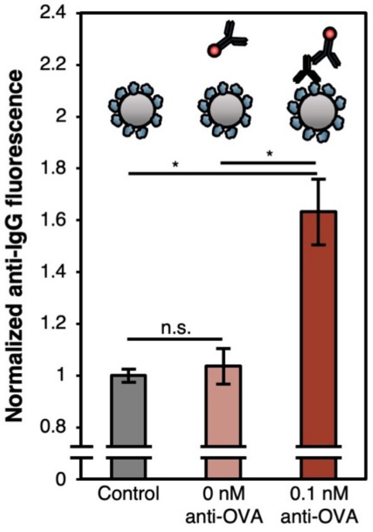

Fig. S5. fNACP detection specificity. fNACP fluorescence intensity of blank fNACPs, fNACPs incubated with only fluorescent anti-IgG, and fNACPs incubated with fluorescent anti-IgG after incubation with 0.1 nM anti-OVA. Data is presented as mean MFI ± standard deviation. N = 3, *P<0.05. Significance between conditions was evaluated using a one-tailed Student's t-test.

Additional pipette details. The printed and assembled acoustic pipette, shown predominantly in schematic form in Fig. 6 of the main text, are shown in Fig. S6.

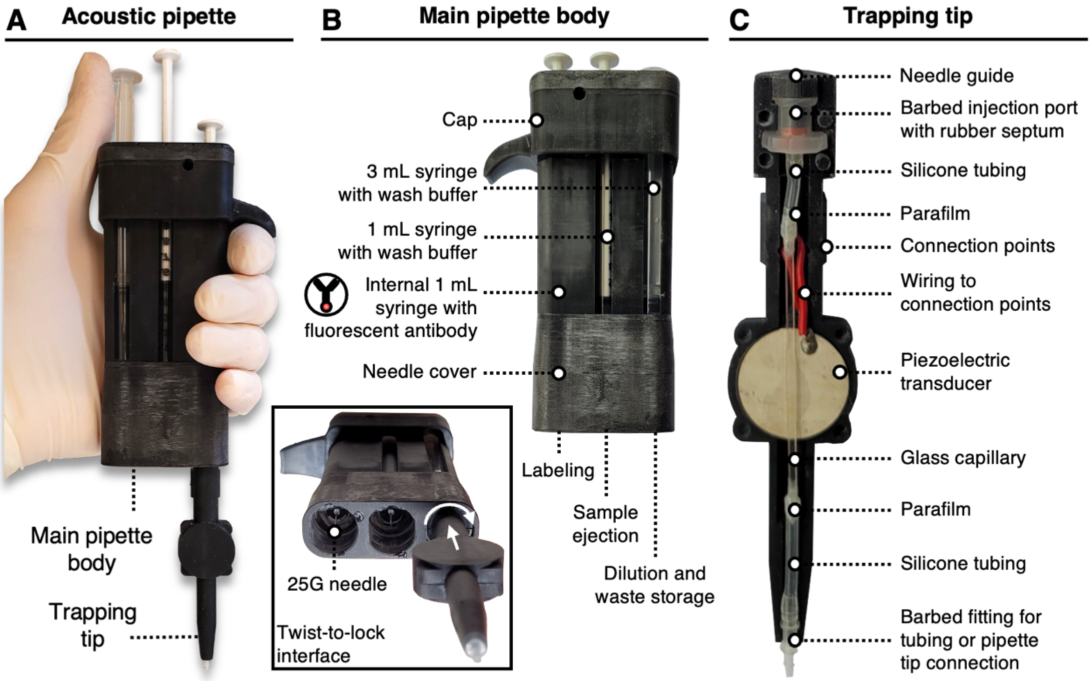

Fig. S6. Additional acoustic pipette overview. (A) Image of acoustic pipette. Inset image shows mechanism for movement and locking of trapping tip, as well as internal needles used to access the trapping channel. **(B)** Image and details of the main pipette body. **(C)** Image and details of the trapping tip.

To enable others to reproduce and utilize the acoustic pipette design, we have included stereolithography (STL) files for each of the 3D printed components of the acoustic pipette (Fig. S7). Using these files, readers may print necessary components using personal 3D printers. Notably, due to differences in printer performance, material, and plating approaches, some minor post-printing modifications may be necessary to enable proper pipette function.

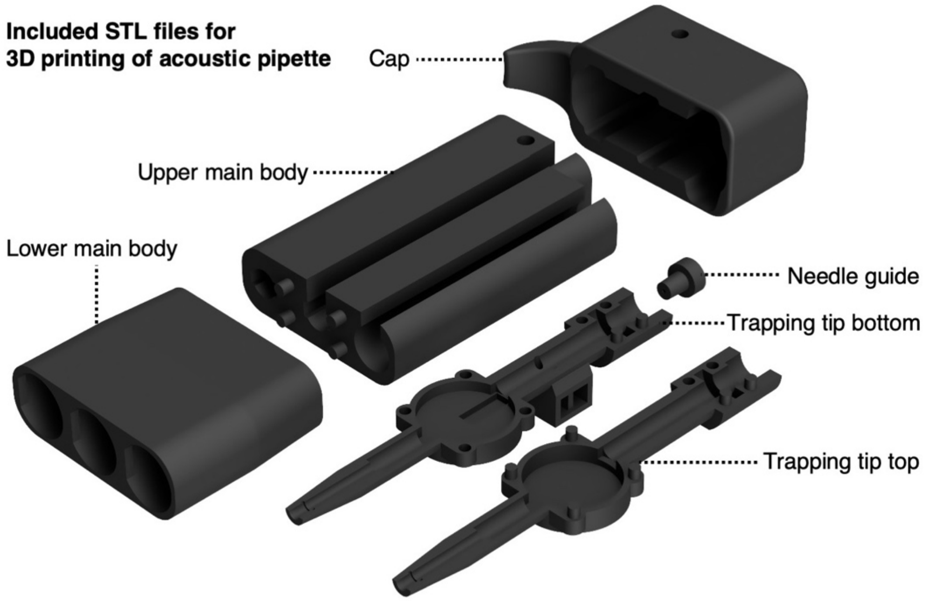

Fig. S7. Acoustic pipette STL files included for 3D printing. 3D renders of STL files for printing of main pipette components.

Additional pipette assembly. Necessary materials, both 3D printed and non-printed, for assembly of the acoustic pipette are detailed in Fig. S8. In addition to the listed components of the pipette (Fig. S8A), assembly requires cyanoacrylate glue, solder, a soldering iron, and wire cutters. To assemble the main body, the upper main body and lower main body are fit together by a pressure fit (Fig. S8B and C). If needed, cyanoacrylate glue can be added to the interface between the two parts to ensure a strong bond. After attaching needles to the 3 mL and 1 mL syringes, the syringes can be inserted into the main body. The cap is then placed over the main body; if needed, a small rod can be inserted through the hold in the cap and upper main body to ensure the cap remains in place. To assemble the trapping channel, a wire should be soldered to each side of the piezoelectric transducer, both slightly off center to allow space for the capillary (Fig. S8D). Then, the glass capillary should be glued to the transducer using a thin layer of cyanoacrylate glue. The capillary should lay flat against the transducer. The silicone tubing can then be attached to each end of the capillary. If needed, the connection can be sealed with cyanoacrylate glue and parafilm. The

silicone tube on the side of the transducer with wires can be connected to a barbed luer lock, into which the rubber septum and needle guide can be inserted. The other silicone tubing can be attached to a barbed tubing connector. With that, the trapping channel components can be placed in the trapping tip bottom, with the wires being routed through the holes to the connection points (Fig. S8E). Finally, the trapping tip top can be pressure fit to the trapping tip bottom to contain the trapping channel components (Fig. S8F).

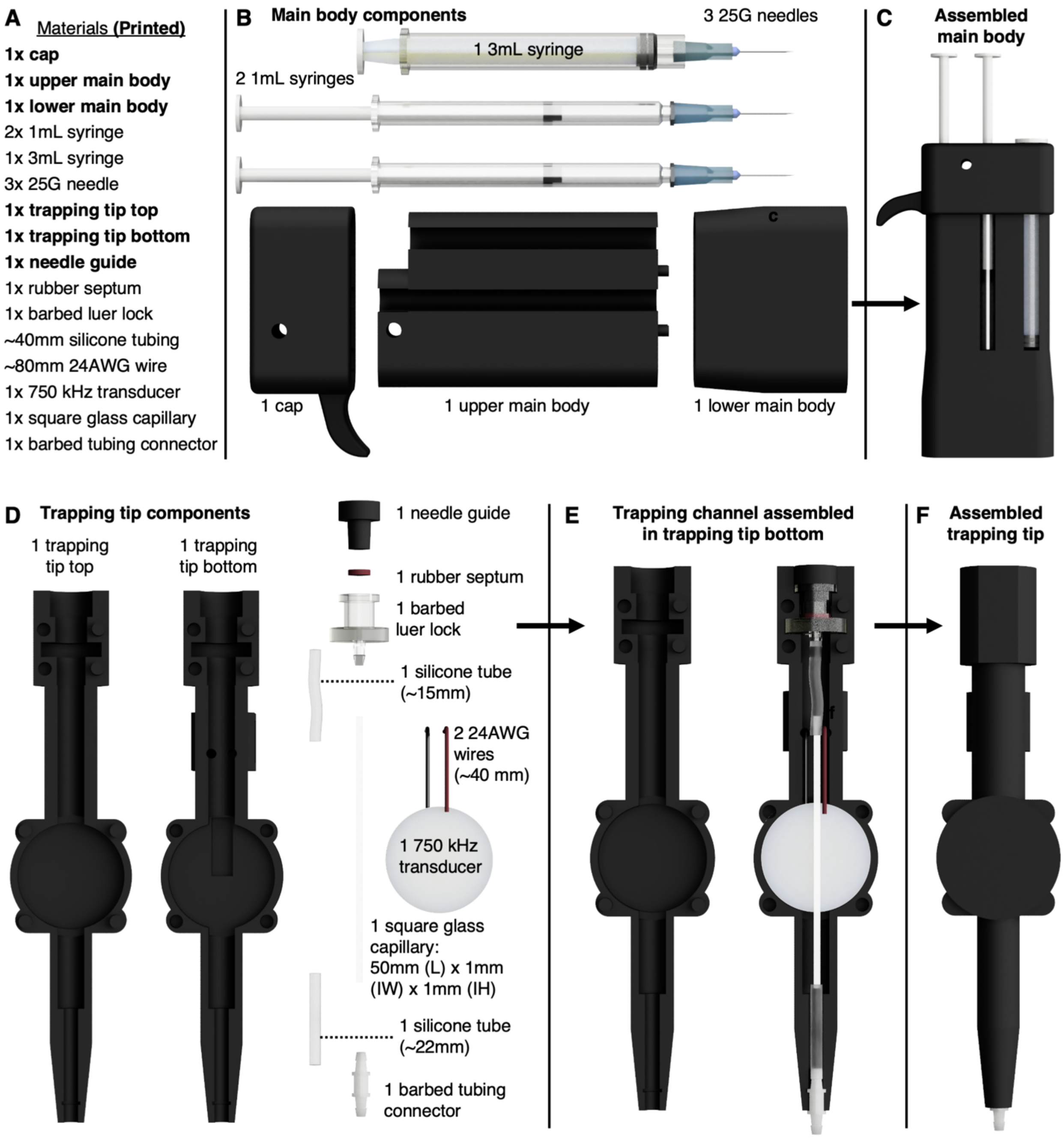

Fig. S8. Materials for acoustic pipette assembly. (A) List of pipette components needed for acoustic pipette assembly, with 3D printed components denoted in bold text. **(B)** Render of disassembled main body components. **(C)** Render of assembled main pipette body. **(D)** Render of disassembled trapping tip components. **(E)** Render of assembled trapping channel in trapping tip bottom. **(F)** Render of assembled trapping tip.

Main fluorimeter housing fabrication and assembly. The fluorimeter structural housing consisted of five 3D-printed structural components and multiple commercially available components (Fig. S9). The five 3D-printed structural components (STLs available in Supplemental Materials) include the main fluorimeter housing, the pipette docking cap, a SP filter holder, a LP filter holder, and a photodiode holder. These components are modified versions of the commercially available Thorlabs CVH100/M (cuvette holder with fiber adapter assembly), CVH100-CV (light-tight cover for cuvette holder), CVH100-FH (mounted filter holder), CP35_M (30 mm Cage Plate with Ø1" Double Bore M4 Tap), and CP33_M (SM1-Threaded 30 mm Cage Plate 0.35" Thick 2 Retaining Rings M4 Tap), respectively. All five structural components were designed using Fusion 360 and printed on a Bambu P1S printer with matte black PLA and a 0.2 mm layer height. The main fluorimeter housing (Fluorimeter main housing.STL) provided structural support for holding the 100 uL cuvette (Thorlabs, CV10Q1FE), pipette docking cap, filters, photodiode, and fiber adapter lens assemblies. One-inch ports with 30 mm rectangularly patterned M2 compatible holes were located on all four sides of the housing, which accepted the LP filter holder and photodiode holder cage plates (Fluorimeter LP holder.STL and Fluorimeter photodiode holder.STL respectively). A Thorlabs SMA905 threaded fiber adapter with 20 mm focal length lens press fit into the front of the housing. Light was provided through a compatible SMA fiber optic cable. The SP filter holder (Fluorimeter SP holder.STL) housed a Thorlabs FESH0600 600 nm SP filter directly in front of the fiber adapter lens. The filter was held in the mount with an M3 4.0 mm setscrew inserted at a 45-degree angle from the mount side. The sample cuvette was lowered onto the cuvette pedestal inside the main housing through a square access port in the top of the housing. Once in place, the pipette docking cap (Fluorimeter pipette docking cap.STL) was pressed onto the top of the housing. An Edmund #15-457 590 nm 1-inch LP filter

was placed into the LP filter holder and locked with an M4 4 mm set screw. A Thorlabs SM1PD1B large area mounted silicon photodiode with 350-1100 nm sensitivity and a grounded anode self-tapped into the photodiode holder. Electrical connectivity to the photodiode amplifier was achieved with a compatible BNC coaxial cable. Ninety degrees relative to the fiber adapter lens, Thorlabs ER1-P4 cage assembly rods were screwed into the main housing using the cage plates for alignment. The cage plates were ordered such that the filter assembly mates to the housing and the photodiode mates to the filter assembly. The cage plates were pressed firmly towards the main housing to block external light and fixed in place on the cage assembly rods with four M4 4 mm set screws per cage plate. A light seal was created by snapping Thorlabs SM1CP2 externally threaded end caps into side ports opposite the photodiode and fiber adapter ports.

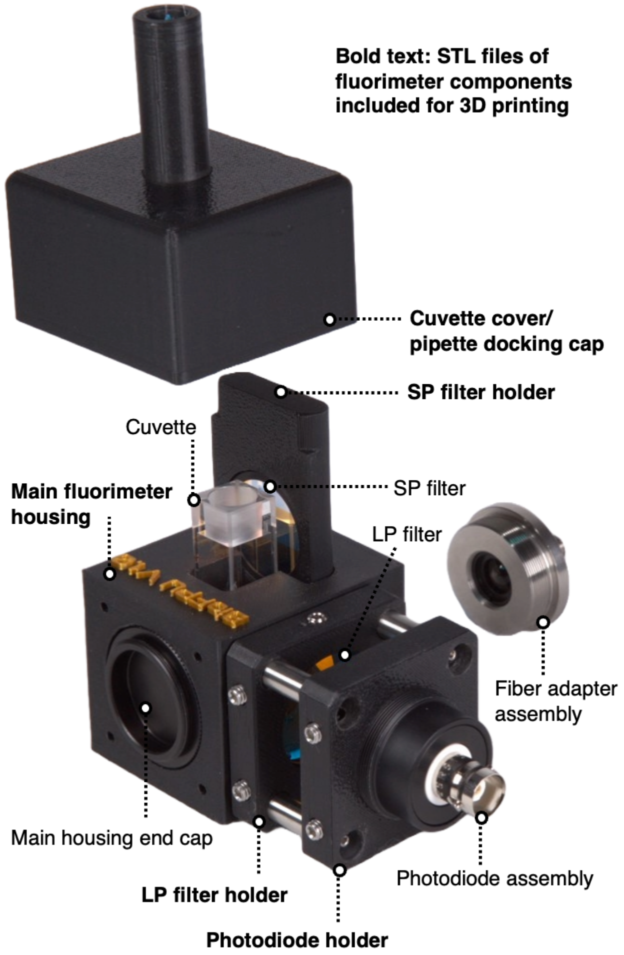

Fig. S9. Exploded view of the main fluorimeter housing and associated components. STL files of all items shown in bold text (the main fluorimeter housing, the pipette docking cap, the SP filter holder, the LP filter holder, and photodiode holder) are available as Supplementary Materials.

Additional fluorimeter cuvette housing details. The cuvette housing accepts one cuvette per experiment. The cuvette used in this work was a 100 uL synthetic quartz cuvette with four polished sides. The sample sat in the bottom of the rectangular channel of the cuvette. Light entered the cuvette housing through the threaded fiber adapter and was focused by the integrated lens before passing through the 600 nm SP filter. This filter removed band light below 610 nm, preventing the

majority of the source light from reaching the photodiode, which would otherwise result in an elevated baseline photocurrent. Filtered light passed through the channel illuminating the sample. The bottom of the cuvette sat 4.0 mm off the housing floor on a pedestal to align the sample channel with the center of the incoming light source, providing maximum illumination of the sample. Light that passed through the cuvette channel terminated on the housing cap opposite to the fiber adapter. To reduce baseline photocurrent, it was important to use non-reflective materials for the end caps to maximize incident light absorption. Light striking the sample causes the fluorophore on fNACP samples to fluoresce, emitting photons with max emission at ~617 nm in all directions. Photons emitted at a right angle to the incoming light first passed through a 590 nm LP filter before striking the silicon photodiode mounted to the housing wall. The LP filter passed light above 610 nm, further attenuating light within the housing that was not generated from the fluorophore while passing the photons from sample fluorescence to the photodiode detector. The large area of the silicon photodiode provided a large target for integrating many photons, which helped to compensate for the moderate photodiode responsivity of ~.35 A/W at 617 nm. Other photodiode technologies like avalanche photodiodes could be used to significantly increase the detector sensitivity, allowing for a lower minimum threshold of detection and reduced demand on the photodiode amplifier performance.

LED and driver. The fluorometer light source was generated by a Thorlabs M565F3 565 nm with 105 nm BW fiber coupled LED, which can be driven with a maximum of 700 mA at a typical forward voltage drop of 2.9 V. Typical emitted light power is 13.5 mW for 1995 mW of electrical power when used with a 400 μm diameter multimode fiber. A threaded optical SMA connector mounted on the LED housing allowed for the mating of an SMA multimode fiber optic cable for

light transmission to the main fluorimeter fiber adapter. For this system, a 1 m long and 400 um diameter SMA-SMA multimode fiber patch cable (Thorlabs, M28L01) was used. The LED was driven with a 1.2 A at 8.0 V (maximum) programmable constant current source (Thorlabs, UPLED) connected by an included Thorlabs M8 x 1 compatible circular connector cable. The LED driver was powered using a Thorlabs DS12 12V AC to DC power supply and connected to a software control GUI (Thorlabs, upSERIES) via a standard data micro-USB cable. The GUI was used to enable and disable power to the LED as well as set the LED drive current. During data collection, the LED was powered for ~30 min at 700 mA to let the device stabilize thermally and optically before any measurements were taken.

Photodiode amplifier and photodiode. A Thorlabs SM1PD1B large area mounted silicon photodiode with a grounded anode served as the fluorimeter detector. The photodiode was capable of detecting 350−1100 nm light and had a peak responsivity of .725 A/W at 970 nm, a maximum dark current of 600 nA, and a junction capacitance of 375 pF measured at 5.0 V. The photodiode had a male BNC connector to electrically interface the diode anode to the cable shield and the cathode to the inner conductor. A generic BNC to BNC cable was used to connect the photodiode to a benchtop photodiode amplifier (Thorlabs, PDA200C). The photodiode amplifier had a full-scale current measurement range from 100 nA to 10 mA with a bandwidth of 1-500 kHz, respectively, with RMS noise of 0.02% of the full-scale range. In all experiments, the amplifier was set to a 100 nA range, which corresponds to a bandwidth of 1 KHz, a resolution of 10 pA, and an analog output conversion coefficient of 10^8 V/A, limited between 0−10 V. Both the amplifier input connected to the photodiode and the output connected to the data acquisition unit accepted a standard BNC connection. Before collecting data, the photodiode amplifier was left running for

one hour to allow it to reach thermal equilibrium. To reduce background signal before experiments, the LED was turned off, the current range was set to 100 nA, and the offset calibration potentiometer was adjusted until a minimum signal output from the photodiode amplifier was recorded on the attached oscilloscope (typically 1.6 mV baseline).

Data acquisition and computer. The analog voltage output of the photodiode amplifier was interfaced to a USB data acquisition unit (Digilent, Analog Discovery 2) with a generic BNC to BNC coaxial cable. The data acquisition unit was connected to a PC using a micro-USB cable. Control software (Digilent, Waveforms) was used to set the scope horizontal and vertical axis to 5 sec/div and 20 mV/div, respectively, with a sampling rate of 100 Hz. For each experiment, the photodiode amplifier voltage was measured for ~50 seconds and the sampled data was recorded to a CSV file for post processing.

5PL fit parameters and validation. Fit parameters for the fits in Figs. 5B and 7B are found in Table S1.

Table S1. 5PL fit parameter values.

Parameter	Fig. 5B	Fig. 7B
a	27.2056	59.733
d	79632	7536.4
c	580.6031	2404600
b	0.765	0.7353
g	0.177	649.9033

After fitting the data in Figs. 5B and 7B, we validated the fits by calculating the average percent relative error (%RE) of experimentally measured values relative to values predicted by the 5PL fits at each anti-OVA concentration investigated. %RE is given by:

$$\%RE = 100\% \times (P - E)/E$$

where P is the predicted MFI and E is the experimentally measured MFI. All values of %RE fell below 25%, and all but one point fell below 13%, suggesting an acceptable fit for both sets of data (Fig. S10).

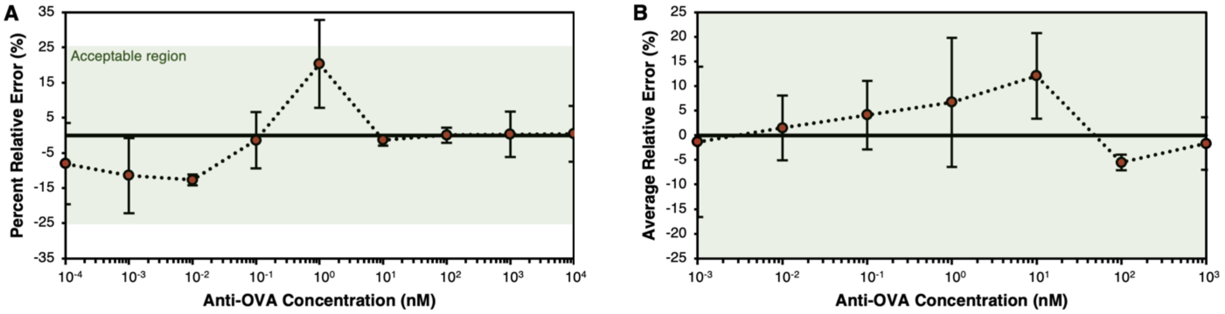

Fig. S10. %RE for 5PL fits. (A) %RE for 5PL fit in Fig. 5B of the main text**. (B)** %RE for 5PL fit in Fig. 7B of the main text**.**

Raw data. The following tables provide raw data used to create plots in the manuscript with N < 20.

Table S2. Raw data for Fig. 3D.

Condition	Retention	Purity
Lysed	0.9467	0.9944
Lysed	0.9907	0.9829
Lysed	0.9973	0.9678
Lysed	0.9946	0.9535
Lysed	0.9983	0.9674
Unlysed	0.9934	0.9300
Unlysed	0.9976	0.9935
Unlysed	0.9948	0.9804
Unlysed	0.9919	0.9785

Table S3. Raw data for Fig. 3E, F, and G.

Flow rate	Retention	Purity
0.5	0.9925	0.9643
0.5	0.9937	0.9757
0.5	1.0000	0.9873
0.5	0.9921	0.9735
0.5	1.0000	0.9938
1	0.9926	0.9655
1	0.9950	0.9759
1	1.0000	0.9090
1	1.0000	0.9779
1	1.0000	0.9881
1.5	1.0000	0.9954
1.5	0.9929	0.9772
1.5	1.0000	0.9878
1.5	0.9964	0.9930
1.5	0.9956	0.9993
2	0.9679	0.9979
2	1.0000	0.9956
2	0.9751	0.9959
2	1.0000	0.9880
2	0.9769	0.9918
3	0.9865	0.9964
3	1.0000	0.9953
3	0.9944	0.9966
3	0.9965	0.9954
3	0.9898	0.9924
5	0.9767	0.9963
5	0.9443	0.9975
5	0.9708	0.9976
5	0.9945	0.9949
5	0.9423	0.9982
6	0.9068	0.9740
6	0.9469	0.9538
6	0.8332	0.9798
6	0.8629	0.9734

Table S4. Raw data for Fig. 3G and S3.

Operator	Retention	Purity
A	0.9025	0.9986
A	1.0000	0.9985
A	0.9800	0.9952
A	0.9799	0.9986
A	0.9451	0.9972
B	0.9903	0.9951
B	0.8441	0.9988
B	0.9985	0.9914
B	0.9976	0.9844
B	0.9818	0.9889
C	1.0000	0.9846
C	0.9906	0.9944
C	0.9923	0.9925
C	1.0000	0.9842
C	1.0000	0.9874
D	0.9973	0.9892
D	1.0000	0.9837
D	0.9890	0.9950
D	1.0000	0.9915
D	0.9976	0.9886
E	0.9975	0.9655
E	0.9526	0.9867
E	0.9850	0.9790
E	0.9974	0.9766

Table S5. Raw data for Fig. 4D.

Condition	NACP FSA Fluorescence (A.U.)
Without separation	17746
Without separation	17692
Without separation	17692
Without separation	18019
Without separation	17855
With separation	17057
With separation	18295
With separation	18129
With separation	18240
With separation	18520

Table S6. Raw data for Fig. 5B.

Anti-OVA Concentration (nM)	Control NACPs Median Anti-IgG Fluorescence (A.U.)	Capture fNACPs Median Anti-IgG Fluorescence (A.U.)
10000	451	28892
10000	373	25891
10000	337	24735
1000	69	11238
1000	80	12771
1000	76	12053
100	42	3146
100	40	3283
100	52	3233
10	34	641
10	41	661
10	34	649
1	34	114
1	31	101
1	35	124
0.1	30	43
0.1	32	48
0.1	34	49
0.01	36	36
0.01	35	35
0.01	35	34
0.001	31	29
0.001	32	29
0.001	38	36
0.0001	38	35
0.0001	32	29
0.0001	29	27
0	33	32
0	32	29
0	31	28
	31	28

Table S7. Raw data for Fig. 7B.

Anti-OVA Concentration (nM)	Control NACPs Median Anti-IgG Fluorescence (A.U.)	Capture fNACPs Median Anti-IgG Fluorescence (A.U.)
1000	258	7203
1000	305	6554
1000	254	6534
100	83	2612
100	101	2581
100	97	2669
10	97	474
10	101	529
10	94	549
1	80	130
1	121	164
1	100	154
0.1	95	80
0.1	84	70
0.1	82	75
0.01	89	67
0.01	78	61
0.01	80	59
0.001	110	77
0.001	103	57
0.001	94	54
0	258	67
0	300	66
0	256	68
0	176	62
0	224	64
0	236	67

Table S8. Raw data for Fig. 7F.

Anti-OVA Concentration (nM)	Capture fNACPs Anti-IgG Fluorescence (A.U.)
1000	1.2215
1000	1.2320
1000	1.2270
100	1.0746
100	1.1171
100	1.0993
10	1.1088
10	1.0510
10	1.0267
0	1.0150
0	1.0332
0	0.9957

Table S9. Raw data for Fig. S5.

Condition	Capture fNACPs Median Anti-IgG Fluorescence (A.U.)
No Secondary	29.3
No Secondary	28.6
No Secondary	27.8
No Primary	31.8
No Primary	29.0
No Primary	28.0
Primary and Secondary	42.5
Primary and Secondary	48.2
Primary and Secondary	49.2

List of additional supplementary files

Supplementary Movie 1: NACP Trapping. NACP trapping in acoustofluidic trapping channel.

Supplementary STL 1: STL file of the AP cap.

Supplementary STL 2: STL file of the AP lower main body.

Supplementary STL 3: STL file of the AP needle guide.

Supplementary STL 4: STL file of the AP trapping tip bottom.

Supplementary STL 5: STL file of the AP trapping tip top.

Supplementary STL 6: STL file of the AP upper main body.

Supplementary STL 7: STL file of the fluorimeter LP holder.

Supplementary STL 8: STL file of the fluorimeter main housing.

Supplementary STL 9: STL file of the fluorimeter photodiode holder.

Supplementary STL 10: STL file of the fluorimeter pipette docking cap.

Supplementary STL 11: STL file of the fluorimeter SP holder.
